# Detection of Porcine–Human Reassortant and Zoonotic Group A Rotaviruses in Humans in Poland

**DOI:** 10.1155/2024/4232389

**Published:** 2024-09-24

**Authors:** Iwona Kozyra, Janusz Kocki, Artur Rzeżutka

**Affiliations:** ^1^ Department of Food and Environmental Virology National Veterinary Research Institute, Al. Partyzantów 57, Puławy 24-100, Poland; ^2^ Department of Medical Genetics Medical University of Lublin, ul. Radziwiłłowska 11, Lublin 20-080, Poland

**Keywords:** human, infection, molecular typing, pig, zoonotic rotavirus

## Abstract

Group A rotaviruses (RVAs) are widespread in humans and many animal species and represent the most epidemiologically important rotavirus group. The aim of the study was the identification of the genotype pattern of human RVA strains circulating in Poland, assessment of their phylogenetic relationships to pig RVAs and identification of reassortant and zoonotic virus strains. Human stool samples which were RVA positive (*n* = 166) were collected from children and adults at the age of 1 month to 74 years with symptoms of diarrhoea. Identification of the G and P genotypes of human RVAs as well as the complete genotype of reassortant and zoonotic virus strains was performed by the use of an RT-PCR method. The G (G1–G4, G8 or G9) and/or P (P[4], P[6], P[8] or P[9]) genotypes were determined for 148 (89.2%) out of 166 RVA strains present in human stool. G1P[8] RVA strains prevailed, and G4P[8] (20.5%), G9P[8] (15.7%) and G2P[4] (13.3%) human RVA strains were also frequently identified. The full genome analysis of human G4P[6] as well as pig G1P[8] and G5P[6] RVAs revealed the occurrence of porcine–human reassortants and zoonotic RVAs. Detection of G4P[6] in pigs confirms their role as a reservoir of zoonotic RVAs.

## 1. Introduction

Group A rotaviruses (RVAs) are widespread in humans and many animal species and represent the most epidemiologically important serological group among rotaviruses [1, 2]. They are non-enveloped viruses containing double-stranded RNA [3]. Particular segments of the genome encode information related to formation of the structural virus proteins (VP1, VP2, VP3, VP4, VP6 and VP7) and depending on the RVA strain, from 5 to 6 non-structural proteins which are involved in virus replication [4, 5]. It is noteworthy that VP4 and VP7 proteins are involved in the infection of the host cell and induction of immune response and are the foundation for classification of RVA strains into the G (VP7) or P (VP4) genotype [6, 7]. The segmented structure of the virus genome enables the occurrence of reassortment events between rotaviruses, which rely on the exchange of viral RNA segments between different strains co-infecting the same host [8]. Reassortment is also facilitated by the presence of animals in close proximity to humans, when gene transfers occur between animal and human RVAs, leading to the emergence of zoonotic virus strains capable of crossing the species barrier. The first reports of human disease caused by pig and human reassortant RVA strains were published in the 1980s [9–11]. In addition, G9P[19] human RVAs containing segments of the pig RVA genome have previously been detected in Latin America and Asia [1, 9, 11–15]. Other strains that crossed the species barrier and subsequently spread in the human population are pig G4P[6] RVAs [16–22]. Pig G3P[6] and G9P[6] RVAs have also caused infections in humans [19, 22–27]. Subsequent phylogenetic analyses of virus strains confirmed their close relationship to pig RVAs, suggesting that animals can be a reservoir of RVAs pathogenic to humans. However, in contrast to data on pig RVA strains circulating in Poland, data on molecular typing of the strains causing infections in humans are scarce; and they do not mirror the real prevalence of RVA strains of particular genotypes [28, 29]. This hinders an assessment of the epidemiological significance of infections resulting from possible zoonotic virus transmission from pigs to humans. The aim of the study was identification of the genotype patterns of human RVA strains circulating in Poland, assessment of their phylogenetic relationships to pig RVAs and identification of reassortant and zoonotic virus strains in relation to their common regional (provincial) occurrence in human and pig hosts.

## 2. Materials and Methods

### 2.1. Sequences of Pig RVAs From Poland

For phylogenetic analyses assessing the relationships between pig and human RVA strains as well as for an identification of reassortant and zoonotic virus strains, use was made of the sequences of RVAs detected in pigs in Poland with characteristic genotype patterns of human RVAs (Table 1).

### 2.2. Stool Samples

Archival human stool samples (*n* = 166) were used in the study which had been collected in the years 2013–2015 from children and adults at the age of 1 month to 74 years with symptoms of diarrhoea. The sick persons came from the same regions (provinces) in which pigs infected with RVA strains of genotypes characteristic of zoonotic virus strains were raised. Samples originated from Mazovia and Kujawy–Pomerania (21 samples), Lublin, Podkarpackie, Pomerania and West Pomerania (20), Wielkopolska (19), Świętokrzyskie (16) and Opole (10) provinces. The RVA ELISA-positive faeces samples were material unused after routine testing of stool samples for the presence of rotavirus antigen by Polish regional medical laboratories. They were obtained with the permission of the relevant laboratory authorities. Samples were provided anonymously; therefore, no patients' personal data or medical records were disclosed. They were stored at a temperature below −20°C until analysis. The local Bioethics Committee approved this study [KE-0254/18/01/2022].

### 2.3. Identification of G and P Genotypes of Human RVA Strains

Viral RNA was extracted from stool using a QIAamp Viral RNA Mini Kit (Qiagen, Hilden, Germany) according to the manufacturer's instructions. Amplification of RVA gene fragments encoding VP7 and VP4 proteins was performed by the RT-PCR method using a OneStep RT-PCR Kit (Qiagen) with the following primer sets: VP7F/VP7R [30], Beg9/End9 [31], Gen-VP4F/Con2 [32, 33] and Con3/Con2 [32]. The protocols for virus detection methods and sequencing of the obtained PCR products as well as for identification of RVA genotypes were previously described in detail by Kozyra [34]. The nucleotide sequences for VP7 and VP4 gene fragments of human RVA strains were deposited in GenBank under the respective accession numbers ON548840–ON548887 and ON600481–ON600526.

### 2.4. Identification of Complete Genotypes of Porcine–Human Reassortants and Zoonotic RVA Strains

When strains were detected in humans of RVA with genotypes G3P[6], G4P[6], G5P[6] and G9P[6], which are typical for zoonotic strains of pig origin, their full genotype corresponding to other genome segments encoding structural (VP1, VP2, VP3 and VP6) and non-structural (NSP1, NSP2, NSP3, NSP4 and NSP5) viral proteins was determined. Additionally, the full genotype was also established for a G1P[8] RVA strain which was unusual for a pig host. Amplification of individual genome fragments such as VP1–VP4 [30], VP6 and NSP1[33], NSP2–NSP4 [35] and NSP4 [36] was carried out using the same RT-PCR protocol as for the VP4 and VP7 fragments, changing only the set of primers and temperature profiles to those previously described. The nucleotide sequences of the particular genome segments of the analysed RVA strains were deposited in the GenBank database under the following accession numbers: OQ989123–OQ989129 (VP1), OQ989130–OQ989136 (VP2), OQ989137–OQ989143 (VP3), OQ989144–OQ989150 (VP6), OQ989151–OQ989157 (NSP1), OQ989158–OQ989164 (NSP2), OQ989165–OQ989171 (NSP3), OQ989172–OQ989178 (NSP4) and OQ989179–OQ989185 (NSP5).

### 2.5. Phylogenetic Analysis

For the assessment of genetic resemblance between human and pig RVA strains having the same genotype, the nucleotide sequences representing a particular virus genotype were subjected to phylogenetic analysis separately. When several human RVA strains exhibiting >99.8% nucleotide sequence identity of the analysed gene fragments were detected, only one strain representing a group of strains of a given genotype was selected for further determination of their phylogenetic relatedness. Additionally, selected sequences of pig and human RVA originating from Europe, Asia and North and South America available from GenBank were included in the analyses. The analysed virus sequences met the criteria of the minimum length and percentage covered by the open reading frame for the particular gene defined by the Rotavirus Classification Working Group (RCWG) [37]. Sequences were grouped and aligned using the MUSCLE alignment software (MEGA 7.0, https://www.megasoftware.net). Subsequently they were analysed using a maximum likelihood method with the Tamura–Nei model produced in MEGA 7.0 [38]. The reliability assessment of the phylogenetic tree topology (by bootstrap resampling) was performed at 1000 replicates, and the phylogenetic relationship between the analysed sequences was considered reliable when the bootstrap value was >70%. The similarity (mutual correspondence) of the nucleotide sequences (sequence identity matrix (SIM)) of the VP4 gene fragments of pig and human RVA strains detected in Poland was determined separately for each genotype using the BioEdit sequence alignment editor programme v. 7.2.5 (Ibis Biosciences, https://www.mbio.ncsu.edu/bioedit/bioedit.html).

### 2.6. Statistical Analyses

The frequency occurrence of RVA strains in humans was estimated by the Clopper–Pearson method using R software [39].

## 3. Results

### 3.1. G and P Genotypes of RVA Strains Detected in Humans

**Figure 1:**
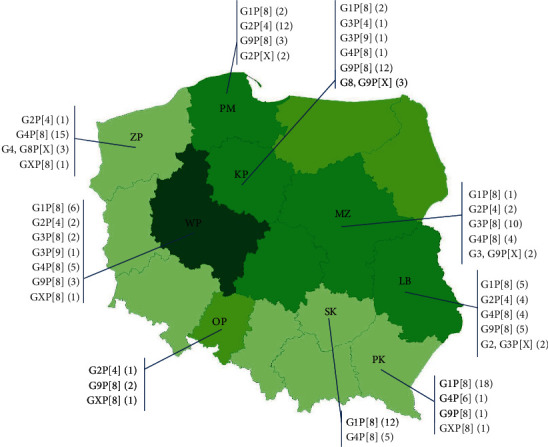
Human RVA strains detected in individual provinces in Poland. KP, Kujawy–Pomerania; LB,Lublin; MZ,Mazovia; OP,Opole; PK,Podkarpackie; PM,Pomerania; SK,Świętokrzyskie; WP,Wielkopolska and ZP,West Pomerania.

Both G and P genotypes were determined for 144 (86.7%) out of 166 RVA strains present in human stool samples, the G genotype was established for 156 (94%) and P for 148 (89.2%) strains. Only one G or P genotype was identified for 16 strains. Infections in humans were caused by RVAs belonging to six G and four P genotypes (Table 2), G1, G4 and P[8] RVAs being the most frequently identified among them. Differences were observed in the frequency of occurrence of G and P RVA genotypes in humans between Polish provinces. The greatest diversity of G genotypes (G1, G2, G3, G4, G8 and G9) among human RVA strains was found in patients in Mazovia, Kujawy–Pomerania and Wielkopolska provinces. In the remaining provinces, mainly virus strains with two or three G genotypes were detected. The most abundant strains, those of P[8] RVA, were prevalent across all provinces covered by the study (Figure 1). Surprisingly, in Świętokrzyskie province strains of this genotype were only found in patients hospitalised due to rotavirus infection. Genotype G1P[8] RVAs were responsible for 27.7% of cases of infection, and their prevalence among infected persons was higher than that of other virus strains. The other frequently identified human RVA strains were G4P[8], G9P[8] and G2P[4] (Table 2).

### 3.2. The Genetic Resemblance Between Human and Pig RVA Strains Determined on the Basis of the Phylogenetic Analysis of VP7 and VP4 Genes

#### 3.2.1. G1 RVA Strains

**Figure 2:**
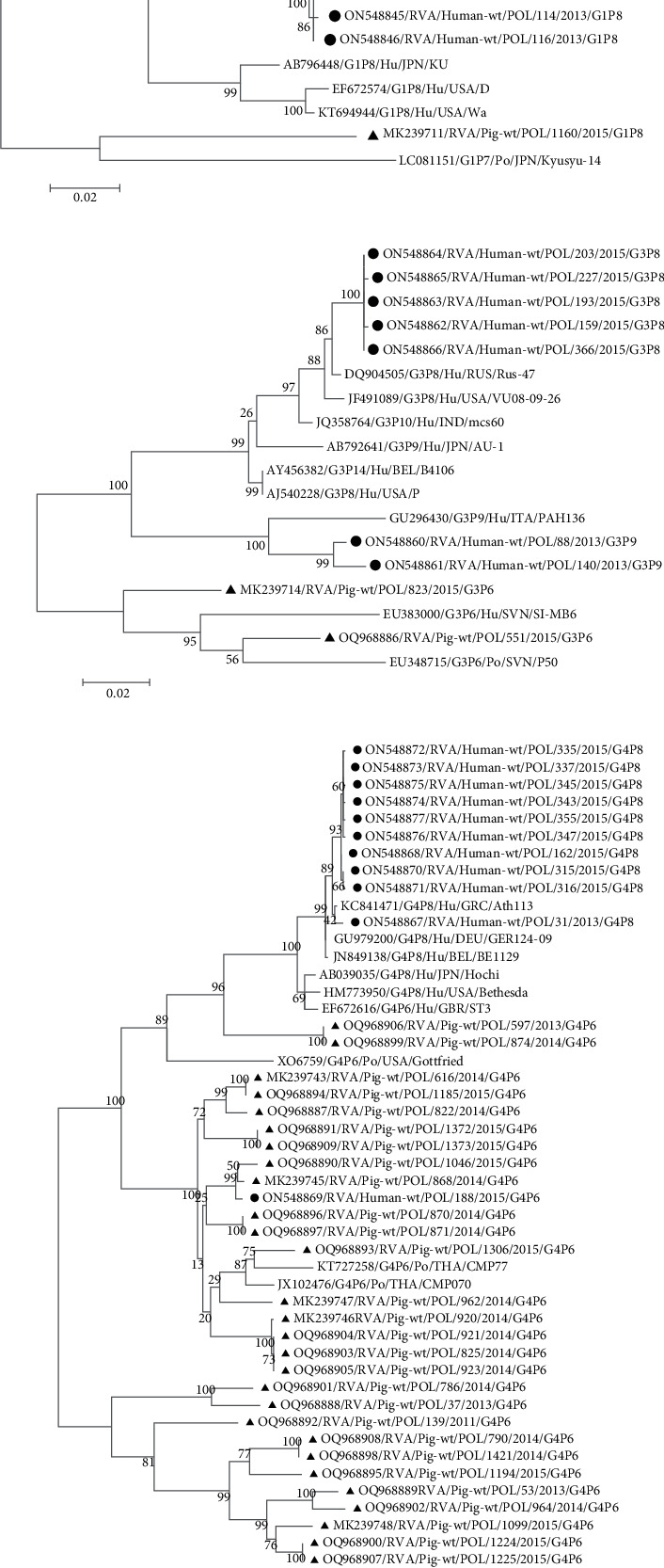
The phylogenetic relationship of G1 (a), G3 (b), G4 (c) and G9 (d) RVA strains circulating in the human and pig populations in Poland and worldwide. The scale bar corresponds to 0.01–0.05 substitutions/nucleotide. The positions on the trees of pig and human RVA strains detected in Poland are marked with a triangle and circle symbol, respectively.

Human G1 RVA strains from Poland showed at least 90% mutual sequence identity (Supporting Information S1: Table 1). While the human-derived strains had relatively high identity, the sequence similarity between human G1 RVAs and pig G1P8/Po/POL/1160 RVA was lower, ranging from 84.1% to 85.1%. The Polish pig RVA strain G1P8/Po/POL/1160 formed a separate clade on the tree from human virus strains (Figure 2a). Other human G1 RVAs which were detected in Poland clustered together with the group of human RVAs circulating in Europe, Asia and North and South America. Four virus strains of human origin (G1P8/Hu/POL/114, G1P8/Hu/POL/116, G1P8/Hu/POL/166 and G1P8/Hu/POL/324) detected in the Lublin, Mazovia, Pomerania and Świętokrzyskie provinces revealed a close genetic similarity to French virus strains responsible for infections in humans (Figure 2a).

#### 3.2.2. G3 RVA Strains

The mutual nucleotide sequence similarity between pig G3P6/Po/POL/551 and G3P6/Po/POL/823 RVA strains was 92.4% (Supporting Information S2: Table 2). In the group of human G3 RVAs detected in Poland, nucleotide sequence resemblance from 89.7% to 100% was observed. The G3 pig and human rotaviruses showed low mutual sequence similarity, not exceeding 88.3%. Nevertheless, Polish pig G3P6/Po/POL/551 and G3P6/Po/POL/823 RVAs were related to the pig and human reassortant G3P6/Hu/SVN/SI-MB6 and to other RVA strains circulating in pigs and humans in Slovenia (Figure 2b).

#### 3.2.3. G4 RVA Strains

The phylogenetic analysis of pig and human G4 RVA strains revealed their mutual genetic resemblance. The G4P6/Hu/POL/188 RVA strain is a porcine–human reassortant example having at least 98.1% nucleotide sequence similarity of its G4 genome fragment to zoonotic pig RVA strains circulating in Poland, but a low 84.9%−85.4% sequence identity with other human G4 RVA strains from Poland (Supporting Information S3: Table 3). The layout of the tree's branches indicates the zoonotic nature of G4P6/Po/POL/868 and G4P6/Po/POL/1046 RVAs, which formed a common cluster with animal rotaviruses (Figure 2c). In addition, three other genetically related pig strains (G4P6/Po/POL/37, G4P6/Po/POL/786 and G4P6/Po/HRV/S400-Vs) could be considered potentially zoonotic, as they were in the same clade as porcine–human reassortant RVA (G4P6/Hu/HUN/BP1227) which caused infections in humans in Hungary. However, on the separate branch but still phylogenetically related was present other porcine–human reassortant strain from Italy which only reviled 83.4% sequence similarity to these pig RVAs.

#### 3.2.4. G9 RVA Strains

Only one RVA strain of human origin (G9P8/Hu/POL/109) was characterised by notable sequence identity with pig RVAs of the same genotype (Supporting Information S4: Table 4), despite forming a separate branch on the phylogenetic tree (Figure 2d).

#### 3.2.5. P[6] RVA Strains

**Figure 3:**
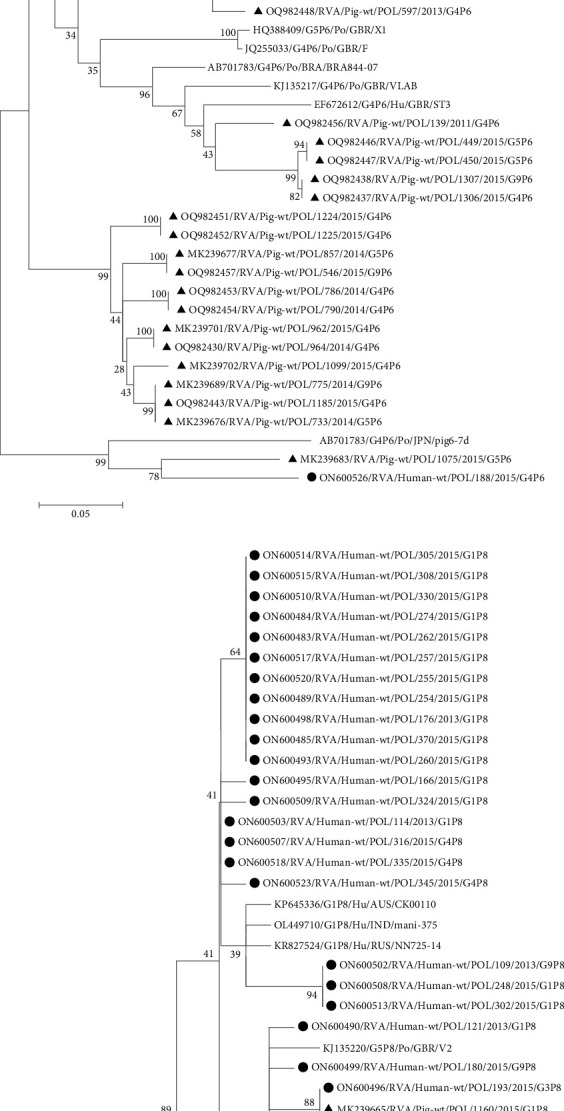
The phylogenetic relationship of P[6] (a) and P[8] (b) RVA strains circulating in the human and pig populations in Poland and worldwide. The scale bar corresponds to 0.02–0.05 substitutions/nucleotide. The positions on the trees of pig and human RVA strains detected in Poland are marked with a triangle and circle symbol, respectively.

The P[6] RVA strains present in the domestic population of pigs revealed higher diversity of the nucleotide sequences of the VP4 gene fragment than that of all other G and P genotypes of pig RVAs. In the studied human population, only one strain of P[6] genotype (G4P6/Hu/POL/188) showed 100% phylogenetic identity with a pig P[6] strain (G5P6/Po/POL/1075 RVA) and confirmed its zoonotic nature. On the other hand, a lower 93%–93.3% genetic resemblance was observed between human G4P6/Hu/ITA/PZ3 zoonotic strain and pig P[6] strains from Poland, which formed a separate clade from other pig P[6] RVAs (Supporting Information S5: Table 5; Figure 3a).

#### 3.2.6. P[8] RVA Strains

The P[8] virus genotype was most often identified among RVA strains circulating in humans rather than in pigs in Poland. The mutual identity of the nucleotide sequences of pig and human P[8] RVA was above 92% (Supporting Information S6: Table 6). The G1P8/Po/POL/1160 RVA strain detected in pigs in Poland showed 99.4%–99.5% genetic resemblance to G1P8/Hu/POL/160 and G1P8/Hu/POL/193 RVAs which caused infections in humans in Mazovia province. The topology of the phylogenetic tree also confirms the common evolutionary origin of pig and human P[8] RVAs by placing pig and human virus strains G1P8/Po/POL/1160, G1P8/Hu/POL/160 and G1P8/Hu/POL/193 from Poland as well as Croatian G1P8/Po/HRV/S372-VS in a common monophyletic group (Figure 3b). Similar phylogenetic relationships were also observed between G1P8/Po/HRV/S441-OB and G9P[8] human strains.

### 3.3. Amino Acid Sequence Analysis of the Structural VP4 Protein of Pig and Human RVA Strains Detected in Poland

The amino acid sequence variability for pig and human P[6] virus strains ranged from 10.7% to 14.6%. Subsequent comparative sequence analysis of zoonotic P[6] virus strains (G4P6/Po/POL/1046 and G5P6/Po/POL/1075) and a representative sequence of G4P6/Po/POL/870 for the entire group of pig P[6] RVAs reviled the presence of 48 amino acid substitutions (Supporting Information S7: Table 7). Common substitutions for zoonotic virus strains were present at positions: 64(I/V), 94 and 95 (I/V), 115 (T/V), 129 (R/K), 133 (I/V), 151 (G/N), 198 (D/N), 203 (V/I) and 229 (I/M). Likewise, in the amino acid sequence of zoonotic G4P6/Hu/POL/188 detected in humans, the variable amino acids sites characteristic for both pig G4P6 and zoonotic pig RVAs were identified at positions 47 (S), 64, 94 and 95 (V), 116 (T), 129 (K), 133 (V), 139 (K), 151 (S), 185 (H), 206 (E), 219 (N), 229(M) and 92 (K), 93 (G), 109 (I), 114(H), 119(D), 129(K), 136(S), 138(D), 153(D) and 245(I), respectively.

By comparing the amino acid sequences of the VP4 protein for G1P8/Po/POL/1160 porcine–human reassortant with other P[8] RVAs circulating in humans in Poland, 21 major amino acid substitutions were observed at the following positions: 30 (T/I), 31 (Q/K), 60 and 72 (T/A), 73 (A/T), 78 (T/S), 85 (T/N), 104 (A/V), 113 (N/D), 130 (V/I), 144 (K/R), 149 (N/S), 162 (R/K), 173 (V/I), 187 (S/G), 194 (D/N), 195 (G/D), 230 (R/I), 234 (P/A), 245 (K/T) and 248 (Q/E) (Supporting Information S8: Table 8). In the group of human P[8] strains there was G1P8/Hu/POL/160 RVA strain which did not reveal any changes in the amino acid sequence within the analysed genome fragment compared to porcine–human G1P8/Po/POL/1160 reassortant strain. In contrast, another human RVA (G1P8/Hu/POL/193) showed only one amino acid substitution at position 162 (V/I).

### 3.4. Molecular Characteristics and Geographical Occurrence of Porcine–Human Reassortants and Zoonotic RVA Strains

Except for the fifth segment of the genome, the segments encoding structural (VP1–VP3 and VP6) and non-structural (NSP1–NSP5) proteins of zoonotic (G4P6/Po/POL/868 and G5P6/Po/POL/1075) strains and the porcine–human reassortant (G4P6/Hu/POL/188) strain had the same genotype pattern. In the case of other human strains, that is, G1P8/Hu/POL/160 and G1P8/Hu/POL/193, as well as the porcine–human reassortant G1P8/Po/POL/1160, the same genotypes were established for 10 viral RNA segments, only the sixth segment being different. A genetic reassortment event between pig and human RVA was also observed for the G1P8/Po/POL/1160 and G5P6/Po/POL/1075 strains (Table 3). The presence of identical genotypes for 10 out of 11 virus genome segments in pig and human RVAs indicated that G4P6/Po/POL/868 and G4P6/Po/POL/1046 RVAs were zoonotic virus strains (Table 3). Interestingly, a G4P[6] RVA strain was identified in humans, although virus strains of this genotype were highly prevalent in pigs in Poland (Table 4). This strain was also detected in pigs kept in the same region (a district in Podkarpackie province) in which a person lived, who suffered from rotavirus diarrhoea caused by a virus strain of the same genotype.

## 4. Discussion

Farm animals can be a source of zoonotic rotavirus strains playing an important role in the epidemiology of rotavirus infections in humans [20, 21]. Phylogenetic analyses of the nucleotide sequences of the gene fragments encoding the VP4 and VP7 proteins of the virus capsid enabled assessment of the strain relatedness and genome sequence divergence resulting from mutations and reassortments of the virus genes [26]. Currently, 42 G and 58 P RVA genotypes have been identified in humans and animals [40]. In this study, RVA strains with G1–G4, G8 and G9 and P[4], P[6], P[8] and P[9] genotype assignments were detected in humans. Among them G1–G4, P[4], P[6] and P[9] have previously been identified in Poland in cases of rotavirus diarrhoea in children [29]. Of note is that virus strains with all of these same genotypes except P[9] have also been detected in pigs in Poland [34]. They have most commonly been found in humans worldwide [2, 41, 42], although G8 and P[9] RVAs have only sporadically been identified in human patients [2, 41, 42]. In this study, there were no significant differences observed in the frequency occurrence among the identified G genotypes of human RVA strains, although G1 RVAs did constitute the most numerous group of strains. Generally in Europe, human infections are mainly caused by G1 RVAs [41, 43–45], while G8 strains have sporadically been detected in animals besides humans [41, 43, 45]. It has been noted that some human G8 RVAs are bovine–human reassortant strains [36, 46]. In Poland, as in other European countries, only single cases of human G8 RVA infections have been detected so far [41, 43, 45]. Four out of the 37 P genotypes identified among human RVAs over the entire world were also found in Poland. Strains of the P[8] genotype dominated, as they were recognised in 76.3% of cases of rotavirus infection. Paralleling the high P[8] RVA prevalence in Poland, strains of this genotype were also frequently noted among human patients in Europe [1, 41, 43–45]. In the group of human RVA strains detected in Poland, there were G4P[6] reassortants containing VP4 and VP7 gene fragments derived from pig RVAs. Strains of this genotype are the most frequently detected zoonotic RVA strains in humans [16–20, 22, 25, 26, 47–54]. Another RVA strain of zoonotic importance besides G4P[6] found in pigs in Poland was G9P[6] RVA. However, it was not detected in humans in this study, although virus strains of the same genotype have been frequently identified in human patients in the United Kingdom, Belgium, the United States of America, Pakistan and India [41, 55–57]. It has been suggested that the presence of RVAs with the same genotype in different hosts, for example, G4P[6], G9P[6] and G9P[8], could indicate strain reassortment events [19–22, 26, 51, 54, 56]. Of note is that zoonotic RVA strains of pig origin are of the P[6] genotype [58, 59]. The ability of pig RVAs to cross the species barrier is mostly associated with the structure of the VP4 protein having binding affinity to sialic acid receptors present in pig intestinal epithelial cells but also present on the surface of human respiratory and urogenital tract cells [58, 60, 61]. However, the incomplete compatibility between the antigenic determinants of P[6] RVA strains and the receptors on human cells prevents the spread of animal virus strains in a human host being rapid [58, 59]. Polish RVA strains affiliated to the G1 and G3 genotypes which could infect both pigs and humans formed phylogenetically closely related clusters which were different to the clades encompassing strains of the same genotypes which were specific to a particular host. However, in the population of pig G3 RVAs there was the G3P6/Po/POL/551 strain showing closer genetic similarity to a Slovenian porcine–human G3 reassortant RVA than to other pig G3 RVA strains [25]. This genetic resemblance could have indicated that in the group of Polish pig G3 RVAs, there are strains which may be considered potentially zoonotic. In general, the zoonotic nature of pig G3 strains has previously been confirmed by cases of human infection in Italy, China and Brazil [24, 51, 62]. The presence of potentially zoonotic strains was also found in the group of pig G9 RVA strains. One, G9P6/Po/POL/775 pig RVA, was in a common phylogenetic cluster with human RVAs. It also showed a closer genetic relationship to human than to pig G9 RVA strains. Another piece of evidence for pigs as a source of infectious rotaviruses for humans in Poland was the detection of G9P8/Hu/POL/109 RVA, having at least 93.4% nucleotide sequence identity of its VP4 genome segment with those of pig G9P[6] RVAs. Nevertheless, pig and human G9 RVA strains showed low genetic variability regardless of their host, and these results are consistent with previous observations [2]. The evolutionary relationship between pig and human G9 RVA has also been confirmed by other studies showing that human strains of this genotype are most likely pig RVAs adapted to the human host [2, 63, 64]. During this present assessment of the genetic relationship between pig and human G4 RVAs originating from Poland, it was shown that they belonged to phylogenetically separate groups. However, in both pig and human hosts, there were not only strains having high mutual genetic resemblance but also zoonotic strains. The analysis of the whole virus genome of zoonotic pig G4P6/Po/POL/868, G4P6/Po/POL/1046 and G5P6/Po/POL/1075 as well as human G4P6/Hu/POL/188 RVA detected in this study revealed that the human strain is a porcine–human reassortant virus. High genetic similarity of the VP7 genome segment at 98.9% was found between human G4P6/Hu/POL/188 RVA and pig G4P6/Po/POL/868. Additionally, the human strain also shared 97.9% nucleotide sequence identity within the same genome segment with pig G4P6/Po/POL/1046 RVA. Both G4P6/Po/POL/868 and G4P6/Po/POL/1046 RVA strains shared the same genotype pattern for particular genome segments, with the exception of the NSP1 protein gene. Furthermore, G4P6/Hu/POL/188 RVA showed a closer genetic resemblance to pig strains than to other human G4 RVA, with which it displayed less than 85.4% similarity. The phylogenetic analysis of the virus VP4 gene also confirmed the common evolutionary origin of pig P[6] and human G4P6/Hu/POL/188 RVA. As with nucleotide sequence analysis also, the presence of variable amino acids sites characteristic for pig and zoonotic pig P[6] RVAs confirms this finding. The transmission of G4-P[6]-I1-R1-C1-M1-A8-N1-T1-E1-H1 RVA from pigs to humans has previously been reported [20, 23]. Similarly, the VP4 gene of pig G5P6/Po/POL/1075 detected in this study revealed a 100% phylogenetic relationship with human G4P6/Hu/POL/188 RVA. It has to be emphasised that G5P[6] RVA strains had not been detected in humans in Poland before this. Neither has more than a single case of human infection caused by RVA of this genotype been reported in Europe [65]. It has been suggested that infections in humans caused by G5P[6] RVAs resulted from direct virus transmission from pigs [65–68]. It is significant that the I1 genotype of G5P6/Po/POL/1075 has been identified in zoonotic pig RVA strains [17, 19, 51]. Evidence of the reassortment events between pig and human RVAs is the emergence of zoonotic G4P6/Hu/POL/188 RVA, the sixth segment (the I1 genotype) of the genome of which originates from pig virus strains [59]. Strains of I1 genotype have also been found in porcine–human virus reassortants causing infections in humans [17, 19, 51]. The identification of this genotype in the population of human RVA strains (G4P6/Hu/POL/188, G1P8/Hu/POL/160 and G1P8/Hu/POL/193) in Poland also indicates the ongoing reassortment events. It is salient that the presence of RVA of the I1 genotype in pigs has only been reported in Belgium so far [59]. However, G4P[6] RVA strains having the same genotype composition determined for all genome segments as zoonotic pig G4P6/Po/POL/868 RVA strains from Poland have been detected in humans in Croatia, Argentina, Paraguay and China [17, 19, 23, 51]. What is more, infections in humans caused by porcine–human G4P[6] RVA reassortants have been widely reported [16–19, 22, 25, 26, 48–51, 53, 54]. A further example of a gene reassortment event between pig and human RVAs is the assignment of the NSP-1 gene of pig G1P8/Po/POL/1160 RVA to the A1 genotype, which is a typical genotype for human RVAs. Strains of the G1P[8] genotype are often detected in humans worldwide, including in Poland, while in pigs they occur only sporadically [41, 69, 70]. In fact, all genotypes of particular genome segments of pig G1P8/Po/POL/1160 RVA, except genotype I5 (segment 6/VP6) are typical for human RVA strains. Likewise, the analysis of VP4 amino acid sequence of porcine–human reasortant G1P8/Po/POL/1160 provides further molecular evidence for its adaptive evolution to pigs. Surprisingly, this analysis revealed the entire concordance of the amino acid profiles between human G1P8/Hu/POL/160 and porcine–human reasortant strain G1P8/Po/POL/1160. In the case of G1P8/Hu/POL/193, only a single amino acid substitution was observed.

Currently, there is no scientific data available on vaccine effectiveness in preventing RVA infections in humans and pigs caused by virus strains holding new genotypes. Therefore, it seems to be advisable to carry out a continuous epidemiological surveillance of infections combined with identification of the virus genotypes. It will allow to assess a real impact of the vaccines used on the appearance and variability of the genetic profile of circulating RVA strains. In contrast to humans, rotavirus vaccines are not considered in immunoprophylaxis programmes of pig herds in Poland, although they are available on the European market. RVAs are ubiquitous microorganisms, therefore efficient protection of pigs against infection is not possible. Usually, RVA infections are characterised by diarrhoea which tends to be severe, especially when *Escherichia coli* co-infections appear. In fact, vaccination may not always provide sufficient protection against RVA infection due to differences observed between the antigenic composition of the virus strains present in the vaccine and the low degree of cross-reactivity of generated antibodies towards the antigenic determinants of the virus strains circulating in the immunised animal population. In Polish pig herds where rotavirus infections have particularly severe course, the use of autovaccines is recommended [71].

A major limitation of this study may be the small number of tested human stool samples and identified virus strains, which may not fully reflect the genotype pattern of the circulating RVAs in humans in Poland. It could also have affected the results of the phylogenetic analyses and the relationships observed between human and pig viruses. Nevertheless, the identified examples still shed light on the evolution and genetic variability of RVAs circulating in human and pig hosts in Poland, providing evidence for their previous genetic reassortment and zoonotic transmission.

## 5. Conclusions

This study showed the genetic diversity among RVAs circulating in humans in Poland. It also provided evidence of the formation of novel and antigenically different virus strains able to cross the species barrier. As zoonotic virus strains emerge, the mechanisms which lead to this can be better recognised and understood by tracking the virus' evolution. The occurrence of G4P[6] and G1P[8] RVA in pigs in Poland confirms the role of this animal species as a reservoir of zoonotic RVAs. The findings of this study could refine epidemiological risk assessment related to human infections caused by animal RVAs and predict the suitability of vaccines used in future public health immunisation programmes.

## Figures and Tables

**Table 1:** Pig RVAs used in the phylogenetic analyses.

Pig RVA	GenBank accession number		Animal		
	VP7	VP4	Age (weeks)	State of health	Origin (province)
G1P[8]/Po/POL/1160	MK239711	MK239665	10	Healthy	West Pomerania
G3P[6]/Po/POL/551	OQ968886	OQ982433	1.5		

G3P[6]/Po/POL/823	MK239714	MK239668	4	Healthy	Lublin

G4P[6]/Po/POL/962	MK239747	MK239701	4	Diarrhoeic	Kujawy-Pomerania
G4P[6]/Po/POL/964	OQ968902	OQ982430	4		

G4P[6]/Po/POL/37	OQ968888	OQ982458	4	Healthy	Lublin
G4P[6]/Po/POL/53	OQ968889	OQ982431	3.5		
G4P[6]/Po/POL/822	OQ968887	OQ982432	4		
G4P[6]/Po/POL/825	OQ968903	OQ982434	6		
G4P[6]/Po/POL/1046	OQ968890	OQ982435	4		

G4P[6]/Po/POL/139	OQ968892	OQ982456	10	Healthy	Mazovia
G4P[6]/Po/POL/1306	OQ968893	OQ982437	5		
G4P[6]/Po/POL/1372	OQ968891	OQ982439	4		
G4P[6]/Po/POL/1373	OQ968909	OQ982440	4		

G4P[6]/Po/POL/1421	OQ968898	OQ982436	3	Healthy	Opole

G4P[6]/Po/POL/921	OQ968904	OQ982441	4	Healthy	Podkarpackie
G4P[6]/Po/POL/923	OQ968905	OQ982442	4		

G4P[6]/Po/POL/920	MK239746	MK239700	4	Diarrhoeic	Podkarpackie

G4P[6]/Po/POL/1185	OQ968894	OQ982443	8	Healthy	Pomerania
G4P[6]/Po/POL/1194	OQ968895	OQ982444	6		

G4P[6]/Po/POL/874	OQ968899	OQ982445	4	Healthy	Świętokrzyskie

G4P[6]/Po/POL/616	MK239743	MK239697	4	Healthy	Wielkopolska
G4P[6]/Po/POL/868	MK239745	MK239699	4		
G4P[6]/Po/POL/871	OQ968897	OQ982450	6		
G4P[6]/Po/POL/1224	OQ968900	OQ982451	8		
G4P[6]/Po/POL/1225	OQ968907	OQ982452	8		

G4P[6]/Po/POL/597	OQ968906	OQ982448	4	Diarrhoeic	Wielkopolska
G4P[6]/Po/POL/870	OQ968896	OQ982449	4		

G4P[6]/Po/POL/786	OQ968901	OQ982453	4	Healthy	West Pomerania
G4P[6]/Po/POL/790	OQ968908	OQ982454	5		
G4P[6]/Po/POL/1099	MK239748	MK239702	2		

G5P[6]/Po/POL/1075	MK239725	MK239683	5	Healthy	Lublin
G5P[6]/Po/POL/733	MK239722	MK239676	7		

G5P[6]/Po/POL/1100	MK239727	MK239680	12	Healthy	Pomerania

G5P[6]/Po/POL/449	OQ968910	OQ982446	20	Healthy	Wielkopolska
G5P[6]/Po/POL/450	OQ968911	OQ982447	20		
G5P[6]/Po/POL/620	MK239720	MK239674	20		

G5P[6]/Po/POL/857	MK239723	MK239677	4	Diarrhoeic	Wielkopolska

G9P[6]/Po/POL/546	OQ968913	OQ982457	5	Healthy	Kujawy-Pomerania

G9P[6]/Po/POL/38	OQ968912	OQ982455	12	Healthy	Lublin

G9P[6]/Po/POL/1040	MK239736	MK239690	5	Healthy	Opole

G9P[6]/Po/POL/775	MK239735	MK239689	6	Healthy	Wielkopolska

G9P[6]/Po/POL/1307	OQ968914	OQ982438	5	Diarrhoeic	Mazovia

**Table 2:** The G/P genotypes of RVA strains detected in humans.

RVA G/P strains (*n* = 166)												
Virus genotype	P[4]		P[6]		P[8]		P[9]		P[X]		Total	
	Number (%)	CI 95%	Number (%)	CI 95%	Number (%)	CI 95%	Number (%)	CI 95%	Number (%)	CI 95%	Number (%)	CI 95%
G1	—	—	—	—	46 (27.7)	21.0–35.2	—	—	—	—	46 (27.7)	21.0–35.2
G2	22 (13.3)	8.5–19.4	—	—	—	—	—	—	3 (1.8)	—	25 (15.1)	10.0–21.4
G3	1 (0.6)	0.01–3.3	—	—	12 (7.2)	3.8–12.3	2 (1.2)	0.1–4.3	2 (1.2)	0.1–4.3	17 (10.2)	6.1–15.9
G4	—	—	1 (0.6)	0.01–3.3	34 (20.5)	14.6–27.4	—	—	1 (0.6)	0.01–3.3	36 (21.7)	17.7–28.7
G8	—	—	—	—	—	—	—	—	3 (1.8)	0.4–5.2	3 (1.8)	0.4–5.2
G9	—	—	—	—	26 (15.7)	10.5–22.1	—	—	3 (1.8)	0.4–5.2	29 (17.5)	12.0–24.1
GX	—	—	—	—	4 (2.4)	0.7–6.0	—	—	6 (3.6)	1.3–7.7	10 (6.0)	2.9–10.8
Total	23 (13.9)	9.0–20.1	1 (0.6)	0.01–3.3	122 (73.5)	66.1–80.0	2 (1.2)	0.1–4.3	18 (10.8)	6.5–16.6	166 (100)	—

*Note:* The numbers in brackets are the detected RVA strains as percentages of all virus strains with at least one G or P genotype identified.

**Table 3:** The complete genotype pattern of zoonotic, porcine–human reassortant and selected human RVA strains.

RVA	Host	Genome segment/Protein coded by gene										
		4/VP7	9/VP4	6/VP6	1/VP1	2/VP2	3/VP3	5/NSP1	8/NSP2	7/NSP3	10/NSP4	11/NSP5
G4P6/Hu/POL/188	Human	G4	P[6]	I1	R1	C1	M1	A1	N1	T1	E1	H1

G4P6/Po/POL/1046	Pig	G4	P[6]	I1	R1	C1	M1	A8	N1	T1	E1	H1

G4P6/Po/POL/868	Pig	G4	P[6]	I1	R1	C1	M1	A8	N1	T1	E1	H1
G5P6/Po/POL/1075		G5	P[6]	I1	R1	C1	M1	A8	N1	T1	E1	H1

G1P8/Hu/POL/160	Human	G1	P[8]	I1	R1	C1	M1	A1	N1	T1	E1	H1
G1P8/Hu/POL/193		G1	P[8]	I1	R1	C1	M1	A1	N1	T1	E1	H1

G1P8/Po/POL/1160	Pig	G1	P[8]	I5	R1	C1	M1	A1	N1	T1	E1	H1

**Table 4:** RVA strains found in pigs and humans in individual Polish provinces.

RVA		Province
Pig	Human	
G4P[6] (*n* = 2) G9P[6] (*n* = 1)	G1P[8] (*n* = 2) G3P[4] (*n* = 1) G3P[9] (*n* = 1) G4P[8] (*n* = 1) G9P[8] (*n* = 12)	Kujawy–Pomerania

G3P[6] (*n* = 1) G4P[6] (*n* = 1) ^*∗*^ G4P[6] (*n* = 3) G5P[6] (*n* = 2) ^*∗*^ G9P[6] (*n* = 1)	G1P[8] (*n* = 5) G2P[4] (*n* = 4) G4P[8] (*n* = 4) G9P[8] (*n* = 5)	Lublin

G4P[6] (*n* = 4) G9P[6] (*n* = 1)	G1P[8] (*n* = 1) G2P[4] (*n* = 2) G3P[8] (*n* = 10) G4P[8] (*n* = 4)	Mazovia

G4P[6] (*n* = 1) G9P[6] (*n* = 1)	G2P[4] (*n* = 1) G9P[8] (*n* = 2)	Opole

G4P[6] (*n* = 3)	G1P[8] (*n* = 18) G4P[6] (*n* = 1) ^*∗*^ G9P[8] (*n* = 1)	Podkarpackie

G4P[6] (*n* = 2) G5P[6] (*n* = 1)	G1P[8] (*n* = 2) G2P[4] (*n* = 12) G9P[8] (*n* = 3)	Pomerania

G4P[6] (*n* = 1)	G1P[8] (*n* = 12) G4P[8] (*n* = 5)	Świętokrzyskie

G4P[6] (*n* = 1) ^*∗*^ G4P[6] (*n* = 6) G5P[6] (*n* = 4) G9P[6] (*n* = 1)	G1P[8] (*n* = 6) G2P[4] (*n* = 2) G3P[8] (*n* = 2) G3P[9] (*n* = 1) G4P[8] (*n* = 5) G9P[8] (*n* = 3)	Wielkopolska

G1P[8] (*n* = 1) ^*∗∗*^ G3P[6] (*n* = 1) G4P[6] (*n* = 3)	G2P[4] (*n* = 1) G4P[8] (*n* = 15)	West Pomerania

^*∗*^Zoonotic pig RVA strains.

^*∗∗*^Porcine–human reassortant RVA strains.

## Data Availability

The data that support the findings of this study are available from the authors upon reasonable request.

## Supporting Information

Supporting Information S1 Table 1: the nucleotide sequence similarity of the VP7 gene fragment of pig and human G1P[8] RVA strains.

Supporting Information S2 Table 2: the nucleotide sequence similarity of the VP7 gene fragment of pig and human G3 RVA strains.

Supporting Information S3 Table 3: the nucleotide sequence similarity of the VP7 gene fragment of pig and human G4 RVA strains.

Supporting Information S4 Table 4: the nucleotide sequence similarity of the VP7 gene fragment of pig and human G9 RVA strains.

Supporting Information S5 Table 5: the nucleotide sequence similarity of the VP4 gene fragment of pig and human P[6] RVA strains.

Supporting Information S6 Table 6: the nucleotide sequence similarity of the VP4 gene fragment of pig and human P[8] RVA strains.

Supporting Information S7 Table 7: the amino acid sequence changes in the VP4 gene fragment of pig and human P[6] RVA strains.

Supporting Information S8 Table 8: the amino acid sequence changes in the VP4 gene fragment of pig and human P[8] RVA strains.
