# Supplementary material for: Detection of Porcine–Human Reassortant and Zoonotic Group A Rotaviruses in Humans in Poland
Source: Transbound Emerg Dis. 2024 Sep 24;2024:4232389. doi: 10.1155/2024/4232389 (PMC12017087; doi:10.1155/2024/4232389)
Supplement: Supporting Information S1 — Table 1: the nucleotide sequence similarity of the VP7 gene fragment of pig and human G1P[8] RVA strains. [file 4232389.f1.pdf]

Supplementary Table S1. The nucleotide sequence similarity of the VP7 gene fragment of pig and human G1P[8] RVA strains

**RVA strain**	G1P8/Po/POL/1160	G1P8/Hu/POL/370	G1P8/Hu/POL/121	G1P8/Hu/POL/260	G1P8/Hu/POL/166	G1P8/Hu/POL/186	G1P8/Hu/POL/114	G1P8/Hu/POL/116	G1P8/Hu/POL/313	G1P8/Hu/POL/302	G1P8/Hu/POL/274	G1P8/Hu/POL/254
G1P8/Po/POL/1160	-	84.4	84.8	84.5	84.9	84.4	84.9	85.1	84.5	84.4	84.5	84.5
G1P8/Hu/POL/370	84.4	-	95.7	99.7	93.3	99.6	93	93.2	99.7	95.7	99.7	99.7
G1P8/Hu/POL/121	84.8	95.7	-	95.7	92.6	95.6	92.4	92.5	95.7	97.8	95.7	95.7
G1P8/Hu/POL/260	84.5	99.7	95.7	-	93.4	99.8	93.2	93.3	99.7	96	100	100
G1P8/Hu/POL/166	84.9	93.3	92.6	93.4	-	93.3	99.4	99.6	93.2	92.1	93.4	93.4
G1P8/Hu/POL/186	84.4	99.6	95.6	99.8	93.3	-	93	93.2	99.6	95.8	99.8	99.8
G1P8/Hu/POL/114	84.9	93	92.4	93.2	99.4	93	-	99.8	92.9	91.8	93.2	93.2
G1P8/Hu/POL/116	**85.1**	93.2	92.5	93.3	99.6	93.2	99.8	-	93	92	93.3	93.3
G1P8/Hu/POL/313	84.5	99.7	95.7	99.7	93.2	99.6	92.9	93	-	95.7	99.7	99.7
G1P8/Hu/POL/302	84.4	95.7	97.8	96	92.1	95.8	91.8	92	95.7	-	96	96
G1P8/Hu/POL/274	84.5	99.7	95.7	100	93.4	99.8	93.2	93.3	99.7	96	-	100
G1P8/Hu/POL/254	84.5	99.7	95.7	100	93.4	99.8	93.2	93.3	99.7	96	100	-
G1P8/Hu/POL/330	84.4	99.8	95.8	99.8	93.3	99.7	93	93.2	99.8	95.8	99.8	99.8
G1P8/Hu/POL/324	84.8	92.6	92	92.8	99	92.6	99.3	99.4	92.5	91.4	92.8	92.8
G1P8/Hu/POL/305	84.4	99.8	95.8	99.8	93.3	99.7	93	93.2	99.8	95.8	99.8	99.8
G1P8/Hu/POL/176	**84.1**	99.6	95.6	99.6	93	99.4	92.8	92.9	99.6	95.6	99.6	99.6
G1P8/Hu/POL/308	84.4	95.7	97.8	96	92.1	95.8	91.8	92	95.7	100	96	96
G1P8/Hu/POL/257	84.5	95.6	97.7	95.8	92	95.7	91.7	91.8	95.6	99.8	95.8	95.8
G1P8/Hu/POL/262	**84.1**	99.6	95.6	99.6	93	99.4	92.8	92.9	99.6	95.6	99.6	99.6
G1P8/Hu/POL/255	84.5	99.7	95.7	100	93.4	99.8	93.2	93.3	99.7	96	100	100
G1P8/Hu/POL/248	84.5	95.6	97.7	95.8	92	95.7	91.7	91.8	95.6	99.8	95.8	95.8
G1P8/Hu/USA/D	85.1	90.2	90.9	90.4	91.6	90.2	91.3	91.4	90.1	90.1	90.4	90.4
G1P8/Hu/BGD/Dhaka16-03	84.9	96.1	98.5	96.1	92.8	96	92.5	92.6	96.1	98.5	96.1	96.1
G1P8/Hu/JPN/KU	85.7	91.2	91.6	91.3	92.2	91.2	92.2	92.4	91.3	91.3	91.3	91.3
G1P8/Hu/USA/Wa	85.1	90.8	91.2	90.9	92.2	90.8	92	92.1	90.6	90.6	90.9	90.9
G1P8/Hu/BRA/IALR113	84.4	94.6	95.2	94.9	93	94.8	93	93.2	94.9	95.2	94.9	94.9
G1P7/Po/JPN/Kyusyu-14	86.1	84.7	85.2	84.8	85.5	84.7	85.2	85.3	84.8	84.4	84.8	84.8
G1P8/Hu/FRA/ADR053-1	84.8	96	99.4	96	92.6	95.8	92.4	92.5	96	98.1	96	96
G1P8/Hu/FRA//E10585	85.7	94.4	93.6	94.5	97.3	94.4	97.3	97.4	94.2	93.3	94.5	94.5
G1P8/Hu/IRL/CIT-H57	85.3	96	98.4	96	92.9	95.8	92.6	92.8	96	98.1	96	96
G1P8/Hu/CAN/BMH-07-011	85.3	96.2	98.6	96.2	92.9	96.1	92.6	92.8	96.2	98.6	96.2	96.2
G1P8/Hu/IRE/R300	84.9	96.4	98.5	96.4	92.9	96.2	92.6	92.8	96.4	98.5	96.4	96.4
G1P8/Hu/SVK/2764	84.4	99.8	95.8	99.8	93.3	99.7	93	93.2	99.8	95.8	99.8	99.8
G1P8/Po/HRV/S372-VS	84.9	95.4	97.7	95.4	91.8	95.3	91.6	91.7	95.4	97.7	95.4	95.4
G1P8/Po/HRV/S440-OB	84.9	95.4	97.7	95.4	91.8	95.3	91.6	91.7	95.4	97.7	95.4	95.4

**RVA strain**	G1P8/Hu/ POL/330	G1P8/Hu/ POL/324	G1P8/Hu/ POL/305	G1P8/Hu/ POL/176	G1P8/Hu/ POL/308	G1P8/Hu/ POL/257	G1P8/Hu/ POL/262	G1P8/Hu/ POL/255	G1P8/Hu/ POL/248	G1P8/Hu/ USA/D	G1P8/Hu/BGD/ Dhaka16-03
G1P8/Po/POL/1160	84.4	84.8	84.4	84.1	84.4	84.5	84.1	84.5	84.5	85.1	84.9
G1P8/Hu/POL/370	99.8	92.6	99.8	99.6	95.7	95.6	99.6	99.7	95.6	90.2	96.1
G1P8/Hu/POL/121	95.8	92	95.8	95.6	97.8	97.7	95.6	95.7	97.7	90.9	98.5
G1P8/Hu/POL/260	99.8	92.8	99.8	99.6	96	95.8	99.6	100	95.8	90.4	96.1
G1P8/Hu/POL/166	93.3	99	93.3	93	92.1	92	93	93.4	92	91.6	92.8
G1P8/Hu/POL/186	99.7	92.6	99.7	99.4	95.8	95.7	99.4	99.8	95.7	90.2	96
G1P8/Hu/POL/114	93	99.3	93	92.8	91.8	91.7	92.8	93.2	91.7	91.3	92.5
G1P8/Hu/POL/116	93.2	99.4	93.2	92.9	92	91.8	92.9	93.3	91.8	91.4	92.6
G1P8/Hu/POL/313	99.8	92.5	99.8	99.6	95.7	95.6	99.6	99.7	95.6	90.1	96.1
G1P8/Hu/POL/302	95.8	91.4	95.8	95.6	100	99.8	95.6	96	99.8	90.1	98.5
G1P8/Hu/POL/274	99.8	92.8	99.8	99.6	96	95.8	99.6	100	95.8	90.4	96.1
G1P8/Hu/POL/254	99.8	92.8	99.8	99.6	96	95.8	99.6	100	95.8	90.4	96.1
G1P8/Hu/POL/330	-	92.6	100	99.7	95.8	95.7	99.7	99.8	95.7	90.2	96.2
G1P8/Hu/POL/324	92.6	-	92.6	92.4	91.4	91.3	92.4	92.8	91.3	91.2	92.1
G1P8/Hu/POL/305	100	92.6	-	99.7	95.8	95.7	99.7	99.8	95.7	90.2	96.2
G1P8/Hu/POL/176	99.7	92.4	99.7	-	95.6	95.4	100	99.6	95.4	90	96
G1P8/Hu/POL/308	95.8	91.4	95.8	95.6	-	99.8	95.6	96	99.8	90.1	98.5
G1P8/Hu/POL/257	95.7	91.3	95.7	95.4	99.8	-	95.4	95.8	100	90	98.4
G1P8/Hu/POL/262	99.7	92.4	99.7	100	95.6	95.4	-	99.6	95.4	90	96
G1P8/Hu/POL/255	99.8	92.8	99.8	99.6	96	95.8	99.6	-	95.8	90.4	96.1
G1P8/Hu/POL/248	95.7	91.3	95.7	95.4	99.8	100	95.4	95.8	-	90	98.4
G1P8/Hu/USA/D	90.2	91.2	90.2	90	90.1	90	90	90.4	90	-	90.5
G1P8/Hu/BGD/Dhaka16-03	96.2	92.1	96.2	96	98.5	98.4	96	96.1	98.4	90.5	-
G1P8/Hu/JPN/KU	91.2	92.1	91.2	90.9	91.3	91.2	90.9	91.3	91.2	96.4	91.7
G1P8/Hu/USA/Wa	90.8	91.8	90.8	90.5	90.6	90.5	90.5	90.9	90.5	99	91
G1P8/Hu/BRA/IALR113	94.8	92.9	94.8	94.5	95.2	95	94.5	94.9	95	91.4	95.6
G1P7/Po/JPN/Kyusyu-14	84.7	85.3	84.7	84.4	84.4	84.5	84.4	84.8	84.5	84.7	84.9
G1P8/Hu/FRA/ADR053-1	96.1	92	96.1	95.8	98.1	98	95.8	96	98	90.4	98.8
G1P8/Hu/FRA//E10585	94.4	96.9	94.4	94.2	93.3	93.2	94.2	94.5	93.2	92.4	93.7
G1P8/Hu/IRL/CIT-H57	96.1	92.2	96.1	95.8	98.1	98	95.8	96	98	90.6	98.8
G1P8/Hu/CAN/BMH-07-011	96.4	92.2	96.4	96.1	98.6	98.5	96.1	96.2	98.5	90.9	99.3
G1P8/Hu/IRE/R300	96.5	92.2	96.5	96.2	98.5	98.4	96.2	96.4	98.4	90.5	99.2
G1P8/Hu/SVK/2764	100	92.6	100	99.7	95.8	95.7	99.7	99.8	95.7	90.2	96.2
G1P8/Po/HRV/S372-VS	95.6	91.2	95.6	95.3	97.7	97.6	95.3	95.4	97.6	90.5	98.4
G1P8/Po/HRV/S440-OB	95.6	91.2	95.6	95.3	97.7	97.6	95.3	95.4	97.6	90.5	98.4

RVA strain	G1P8/Hu/JPN/KU	G1P8/Hu/USA/Wa	G1P8/Hu/BRA/IALR113	G1P7/Po/JPN/Kyusyu-14	G1P8/Hu/FRA/ADR053-1	G1P8/Hu/FRA/E10585	G1P8/Hu/IRL/CIT-H57	G1P8/Hu/CAN/BMH-07-011	G1P8/Hu/IRE/R300
G1P8/Po/POL/1160	85.7	85.1	84.4	86.1	84.8	85.7	85.3	85.3	84.9
G1P8/Hu/POL/370	91.2	90.8	94.6	84.7	96	94.4	96	96.2	96.4
G1P8/Hu/POL/121	91.6	91.2	95.2	85.2	99.4	93.6	98.4	98.6	98.5
G1P8/Hu/POL/260	91.3	90.9	94.9	84.8	96	94.5	96	96.2	96.4
G1P8/Hu/POL/166	92.2	92.2	93	85.5	92.6	97.3	92.9	92.9	92.9
G1P8/Hu/POL/186	91.2	90.8	94.8	84.7	95.8	94.4	95.8	96.1	96.2
G1P8/Hu/POL/114	92.2	92	93	85.2	92.4	97.3	92.6	92.6	92.6
G1P8/Hu/POL/116	92.4	92.1	93.2	85.3	92.5	97.4	92.8	92.8	92.8
G1P8/Hu/POL/313	91.3	90.6	94.9	84.8	96	94.2	96	96.2	96.4
G1P8/Hu/POL/302	91.3	90.6	95.2	84.4	98.1	93.3	98.1	98.6	98.5
G1P8/Hu/POL/274	91.3	90.9	94.9	84.8	96	94.5	96	96.2	96.4
G1P8/Hu/POL/254	91.3	90.9	94.9	84.8	96	94.5	96	96.2	96.4
G1P8/Hu/POL/330	91.2	90.8	94.8	84.7	96.1	94.4	96.1	96.4	96.5
G1P8/Hu/POL/324	92.1	91.8	92.9	85.3	92	96.9	92.2	92.2	92.2
G1P8/Hu/POL/305	91.2	90.8	94.8	84.7	96.1	94.4	96.1	96.4	96.5
G1P8/Hu/POL/176	90.9	90.5	94.5	84.4	95.8	94.2	95.8	96.1	96.2
G1P8/Hu/POL/308	91.3	90.6	95.2	84.4	98.1	93.3	98.1	98.6	98.5
G1P8/Hu/POL/257	91.2	90.5	95	84.5	98	93.2	98	98.5	98.4
G1P8/Hu/POL/262	90.9	90.5	94.5	84.4	95.8	94.2	95.8	96.1	96.2
G1P8/Hu/POL/255	91.3	90.9	94.9	84.8	96	94.5	96	96.2	96.4
G1P8/Hu/POL/248	91.2	90.5	95	84.5	98	93.2	98	98.5	98.4
G1P8/Hu/USA/D	96.4	99	91.4	84.7	90.4	92.4	90.6	90.9	90.5
G1P8/Hu/BGD/Dhaka16-03	91.7	91	95.6	84.9	98.8	93.7	98.8	99.3	99.2
G1P8/Hu/JPN/KU	-	96.8	92.8	85.2	91.6	93.6	91.8	92.1	91.7
G1P8/Hu/USA/Wa	96.8	-	91.8	84.8	90.9	92.8	91.2	91.4	91
G1P8/Hu/BRA/IALR113	92.8	91.8	-	84.9	95.2	94.5	95.7	95.7	95.7
G1P7/Po/JPN/Kyusyu-14	85.2	84.8	84.9	-	85.1	85.7	85.1	84.8	84.9
G1P8/Hu/FRA/ADR053-1	91.6	90.9	95.2	85.1	-	93.6	98.6	98.9	98.8
G1P8/Hu/FRA/E10585	93.6	92.8	94.5	85.7	93.6	-	93.8	94.1	94.1
G1P8/Hu/IRL/CIT-H57	91.8	91.2	95.7	85.1	98.6	93.8	-	98.9	98.8
G1P8/Hu/CAN/BMH-07-011	92.1	91.4	95.7	84.8	98.9	94.1	98.9	-	99.3
G1P8/Hu/IRE/R300	91.7	91	95.7	84.9	98.8	94.1	98.8	99.3	-
G1P8/Hu/SVK/2764	91.2	90.8	94.8	84.7	96.1	94.4	96.1	96.4	96.5
G1P8/Po/HRV/S372-VS	91.7	91	94.9	85.2	98	93	98.4	98.5	98.6
G1P8/Po/HRV/S440-OB	91.7	91	94.9	85.2	98	93	98.4	98.5	98.6

**RVA strain**	G1P8/Hu/SVK/2764	G1P8/Po/HRV/S372-VS	G1P8/Po/HRV/S440-OB
G1P8/Po/POL/1160	84.4	84.9	84.9
G1P8/Hu/POL/370	99.8	95.4	95.4
G1P8/Hu/POL/121	95.8	97.7	97.7
G1P8/Hu/POL/260	99.8	95.4	95.4
G1P8/Hu/POL/166	93.3	91.8	91.8
G1P8/Hu/POL/186	99.7	95.3	95.3
G1P8/Hu/POL/114	93	91.6	91.6
G1P8/Hu/POL/116	93.2	91.7	91.7
G1P8/Hu/POL/313	99.8	95.4	95.4
G1P8/Hu/POL/302	95.8	97.7	97.7
G1P8/Hu/POL/274	99.8	95.4	95.4
G1P8/Hu/POL/254	99.8	95.4	95.4
G1P8/Hu/POL/330	100	95.6	95.6
G1P8/Hu/POL/324	92.6	91.2	91.2
G1P8/Hu/POL/305	100	95.6	95.6
G1P8/Hu/POL/176	99.7	95.3	95.3
G1P8/Hu/POL/308	95.8	97.7	97.7
G1P8/Hu/POL/257	95.7	97.6	97.6
G1P8/Hu/POL/262	99.7	95.3	95.3
G1P8/Hu/POL/255	99.8	95.4	95.4
G1P8/Hu/POL/248	95.7	97.6	97.6
G1P8/Hu/USA/D	90.2	90.5	90.5
G1P8/Hu/BGD/Dhaka16-03	96.2	98.4	98.4
G1P8/Hu/JPN/KU	91.2	91.7	91.7
G1P8/Hu/USA/Wa	90.8	91	91
G1P8/Hu/BRA/IALR113	94.8	94.9	94.9
G1P7/Po/JPN/Kyusyu-14	84.7	85.2	85.2
G1P8/Hu/FRA/ADR053-1	96.1	98	98
G1P8/Hu/FRA/E10585	94.4	93	93
G1P8/Hu/IRL/CIT-H57	96.1	98.4	98.4
G1P8/Hu/CAN/BMH-07-011	96.4	98.5	98.5
G1P8/Hu/IRE/R300	96.5	98.6	98.6
G1P8/Hu/SVK/2764	-	95.6	95.6
G1P8/Po/HRV/S372-VS	95.6	-	100
G1P8/Po/HRV/S440-OB	95.6	100	-
