# Supplementary material for: Detection of Porcine–Human Reassortant and Zoonotic Group A Rotaviruses in Humans in Poland
Source: Transbound Emerg Dis. 2024 Sep 24;2024:4232389. doi: 10.1155/2024/4232389 (PMC12017087; doi:10.1155/2024/4232389)
Supplement: Supporting Information S2 — Table 2: the nucleotide sequence similarity of the VP7 gene fragment of pig and human G3 RVA strains. [file 4232389.f2.pdf]

Supplementary Table S2. The nucleotide sequence similarity of the VP7 gene fragment of pig and human G3 RVA strains

**RVA strain**	G3P9/Hu/POL/88	G3P8/Hu/POL/159	G3P8/Hu/POL/193	G3P8/Hu/POL/203	G3P8/Hu/POL/227	G3P9/Hu/POL/140	G3P8/Hu/POL/366	G3P6/Po/POL/551	G3P6/Po/POL/979	G3P14/Hu/BEL/B4106	G3P9/Hu/JPN/AU-1	TA/PAH136	G3P6/Po/SVN/SI-P50	G3P6/Hu/SVN/SI-MB6	G3P8/Hu/RUS/Rus-47	G3P8/Hu/USA/P	G3P10/Hu/IND/mcs60	G3P8/Hu/USA/VU08-09-26
G3P9/Hu/POL/88	-	90.1	90.2	90.2	90.1	98.6	90.2	86.3	88.1	90.9	90.2	94.6	86.6	86	89.8	90.9	90.1	90.1
G3P8/Hu/POL/159	90.1	-	99.8	99.8	99.7	**89.7**	99.8	87.1	88.3	96.4	95.2	88.6	87.7	87	98.6	96.4	97.5	98.1
G3P8/Hu/POL/193	90.2	99.8	-	100	99.8	89.8	100	87	88.2	96.6	95.4	88.7	87.5	86.9	98.7	96.6	97.7	98.2
G3P8/Hu/POL/203	90.2	99.8	**100**	-	99.8	89.8	100	87	88.2	96.6	95.4	88.7	87.5	86.9	98.7	96.6	97.7	98.2
G3P8/Hu/POL/227	90.1	99.7	99.8	99.8	-	**89.7**	99.8	86.9	88.1	96.4	95.2	88.6	87.4	86.7	98.6	96.4	97.5	98.1
G3P9/Hu/POL/140	98.6	**89.7**	89.8	89.8	**89.7**	-	89.8	86.2	87.7	90.5	89.8	94.1	86.5	86	89.4	90.5	89.7	89.7
G3P8/Hu/POL/366	90.2	99.8	100	100	99.8	89.8	-	87	88.2	96.6	95.4	88.7	87.5	86.9	98.7	96.6	97.7	98.2
G3P6/Po/POL/551	86.3	87.1	87	87	86.9	86.2	87	-	**92.4**	88.3	88.3	86.3	93.9	92.9	87.3	88.3	87	86.7
G3P6/Po/POL/823	88.1	**88.3**	88.2	88.2	88.1	87.7	88.2	92.4	-	89.6	88.7	87.3	91.4	90.8	88.5	89.6	88.2	88.2
G3P14/Hu/BEL/B4106	90.9	96.4	96.6	96.6	96.4	90.5	96.6	88.3	89.6	-	97.4	90	88.3	87.8	97.3	100	97.8	97.3
G3P9/Hu/JPN/AU-1	90.2	95.2	95.4	95.4	95.2	89.8	95.4	88.3	88.7	97.4	-	89.7	87.7	87.3	95.8	97.4	96.4	95.8
G3P9/Hu/ITA/PAH136	94.6	88.6	88.7	88.7	88.6	94.1	88.7	86.3	87.3	90	89.7	-	86.3	85.9	88.9	90	88.6	88.9
G3P6/Po/SVN/SI-P50	86.6	87.7	87.5	87.5	87.4	86.5	87.5	93.9	91.4	88.3	87.7	86.3	-	91.3	87.9	88.3	87.1	87.4
G3P6/Hu/SVN/SI-MB6	86	87	86.9	86.9	86.7	86	86.9	92.9	90.8	87.8	87.3	85.9	91.3	-	86.9	87.8	86.3	86.3
G3P8/Hu/RUS/Rus-47	89.8	98.6	98.7	98.7	98.6	89.4	98.7	87.3	88.5	97.3	95.8	88.9	87.9	86.9	-	97.3	98.3	98.9
G3P8/Hu/USA/P	90.9	96.4	96.6	96.6	96.4	90.5	96.6	88.3	89.6	100	97.4	90	88.3	87.8	97.3	-	97.8	97.3
G3P10/Hu/IND/mcs60	90.1	97.5	97.7	97.7	97.5	89.7	97.7	87	88.2	97.8	96.4	88.6	87.1	86.3	98.3	97.8	-	98.3
G3P8/Hu/USA/VU08-09-26	90.1	98.1	98.2	98.2	98.1	89.7	98.2	86.7	88.2	97.3	95.8	88.9	87.4	86.3	98.9	97.3	98.3	-
