# Supplementary material for: Detection of Porcine–Human Reassortant and Zoonotic Group A Rotaviruses in Humans in Poland
Source: Transbound Emerg Dis. 2024 Sep 24;2024:4232389. doi: 10.1155/2024/4232389 (PMC12017087; doi:10.1155/2024/4232389)
Supplement: Supporting Information S3 — Table 3: the nucleotide sequence similarity of the VP7 gene fragment of pig and human G4 RVA strains. [file 4232389.f3.pdf]

Supplementary Table S3. The nucleotide sequence similarity of the VP7 gene fragment of pig and human G4 RVA strains

**RVA strain**	G4P6/Po/POL/962	G4P6/Po/POL/53	G4P6/Po/POL/1046	G4P6/Po/POL/920	G4P6/Po/POL/616	G4P6/Po/POL/1194	G4P6/Po/POL/868	G4P6/Po/POL/1224	G4P6/Po/POL/786	G4P6/Po/POL/1099	G4P6/Po/POL/37	G4P6/Po/POL/139	G4P6/Po/POL/1306	G4P6/Po/POL/1185	G4P6/Po/POL/964	G4P6/Po/POL/1372	G4P6/Po/POL/822	G4P6/Po/POL/870	G4P6/Po/POL/790
G4P6/Po/POL/962	-	82.5	93.3	93.1	93.5	83,0	93.8	83.4	80.6	83.3	81.3	82.8	93,0	93.5	82.3	92.3	93.1	93.4	82.7
G4P6/Po/POL/53	82.5	-	82.7	83.2	82.7	89.7	82.9	93.4	84,0	93.4	83.3	85.7	82.6	82.7	96.3	81.6	82.7	83.6	90.7
G4P6/Po/POL/1046	93.3	82.7	-	92.8	93.4	82.5	98.2	83.2	82.3	82.9	82.3	83.5	93.4	93.4	82.9	92.7	93.4	94.4	82.2
G4P6/Po/POL/920	93.1	83.2	92.8	-	92.7	83.3	94,0	83.6	81.7	83.2	81.7	82.5	91.6	92.7	83,0	92,0	93.3	93,0	81.5
G4P6/Po/POL/616	93.5	82.7	93.4	92.7	-	82.6	94.5	82.6	81.9	82.5	82.7	84.3	92.1	100,0	82.3	94.4	97.4	94.1	82.3
G4P6/Po/POL/1194	83,0	89.7	82.5	83.3	82.6	-	82.7	91.4	85,0	91,0	84.7	88,0	81.9	82.6	89.6	81.3	83.2	82.5	93.4
G4P6/Po/POL/868	93.8	82.9	98.2	94,0	94.5	82.7	-	83.2	82.3	82.9	82.9	83.5	93.4	94.5	83,0	93.5	94.8	95.3	81.9
G4P6/Po/POL/1224	83.4	93.4	83.2	83.6	82.6	91.4	83.2	-	83.9	95.8	83.9	87.7	82.3	82.6	93,0	81,0	82.7	83.4	91.6
G4P6/Po/POL/786	80.6	84,0	82.3	81.7	81.9	85,0	82.3	83.9	-	84,0	94.3	86,0	82.3	81.9	83.7	82.3	82.6	82.3	86.2
G4P6/Po/POL/1099	83.3	93.4	82.9	83.2	82.5	91,0	82.9	95.8	84,0	-	84,0	87.3	81.9	82.5	92.8	80.9	82.3	83,0	91.6
G4P6/Po/POL/37	81.3	83.3	82.3	81.7	82.7	84.7	82.9	83.9	94.3	84,0	-	85.9	80.7	82.7	83.6	82.5	82.9	83,0	86.3
G4P6/Po/POL/139	82.8	85.7	83.5	82.5	84.3	88,0	83.5	87.7	86,0	87.3	85.9	-	81.9	84.3	86.2	82.1	84.3	83.5	87.2
G4P6/Po/POL/1306	93,0	82.6	93.4	91.6	92.1	81.9	93.4	82.3	82.3	81.9	80.7	81.9	-	92.1	82.5	92.1	92.3	92.4	81.7
G4P6/Po/POL/1185	93.5	82.7	93.4	92.7	100,0	82.6	94.5	82.6	81.9	82.5	82.7	84.3	92.1	-	82.3	94.4	97.4	94.1	82.3
G4P6/Po/POL/964	82.3	96.3	82.9	83,0	82.3	89.6	83,0	93,0	83.7	92.8	83.6	86.2	82.5	82.3	-	81.6	82.3	82.9	90.6
G4P6/Po/POL/1372	92.3	81.6	92.7	92,0	94.4	81.3	93.5	81,0	82.3	80.9	82.5	82.1	92.1	94.4	81.6	-	94.3	93.7	81.3
G4P6/Po/POL/822	93.1	82.7	93.4	93.3	97.4	83.2	94.8	82.7	82.6	82.3	82.9	84.3	92.3	97.4	82.3	94.3	-	94.1	82,0
G4P6/Po/POL/870	93.4	83.6	94.4	93,0	94.1	82.5	95.3	83.4	82.3	83,0	83,0	83.5	92.4	94.1	82.9	93.7	94.1	-	82.2
G4P6/Po/POL/790	82.7	90.7	82.2	81.5	82.3	93.4	81.9	91.6	86.2	91.6	86.3	87.2	81.7	82.3	90.6	81.3	82,0	82.2	-
G4P6/Po/POL/597	84.3	82.3	85.3	84.4	85.4	82.5	85.7	82,0	81.7	81.6	82.3	82.8	85,0	85.4	82,0	84.7	85.4	84.7	82.3
G4P6/Po/POL/825	93.1	83.2	92.8	99.7	92.7	83.3	94,0	83.6	81.7	83.2	81.7	82.5	91.6	92.7	83,0	92,0	93.3	93,0	81.5
G4P6/Po/POL/921	93.1	83.2	92.8	99.7	92.7	83.3	94,0	83.6	81.7	83.2	81.7	82.5	91.6	92.7	83,0	92,0	93.3	93,0	81.5
G4P6/Po/POL/923	93.1	83.2	92.8	99.7	92.7	83.3	94,0	83.6	81.7	83.2	81.7	82.5	91.6	92.7	83,0	92,0	93.3	93,0	81.5
G4P6/Po/POL/1373	92.3	81.6	92.7	92,0	94.4	81.3	93.5	81,0	82.3	80.9	82.5	82.1	92.1	94.4	81.6	100,0	94.3	93.7	81.3
G4P6/Po/POL/871	93.4	83.6	94.4	93,0	94.1	82.5	95.3	83.4	82.3	83,0	83,0	83.5	92.4	94.1	82.9	93.7	94.1	100,0	82.2
G4P6/Po/POL/1421	82.9	90.8	82.3	81.6	82.5	93.5	82,0	91.7	86.3	91.7	86.4	87.3	81.9	82.5	90.7	81.5	82.2	82.3	99.8
G4P6/Po/POL/874	84.3	82.3	85.3	84.4	85.4	82.5	85.7	82,0	81.7	81.6	82.3	82.8	85,0	85.4	82,0	84.7	85.4	84.7	82.3
G4P6/Po/POL/1225	83.4	93.4	83.2	83.6	82.6	91.4	83.2	100,0	83.9	95.8	83.9	87.7	82.3	82.6	93,0	81,0	82.7	83.4	91.6
G4P6/Po/THA/CMP070	93.4	82.7	93.7	92.3	93.8	82,0	94.5	82.2	82.5	81.7	81.9	82.3	95.1	93.8	82.3	93.4	94,0	94.1	81.6
G4P8/Hu/GRC/Ath113	84.9	84.2	85.7	84,0	84.7	83,0	85.6	84.2	82.2	83.4	81.9	81.2	84.4	84.7	83.9	83.3	84.4	84.3	85,0
G4P8/Hu/BEL/BE1129	85.2	83.9	85.7	84,0	84.6	83,0	85.6	84.2	81.9	83.4	81.7	81.2	84.4	84.6	83.6	83.3	84.3	84.9	85,0
G4P8/Hu/USA/Bethesda	85.4	84.2	86.6	84.9	85.7	84.2	86.7	83.9	82.2	83.7	81.7	81.6	85.3	85.7	84.2	84.4	85,0	84.6	84.7
G4P8/Hu/DEU/GER124-09	85.3	84,0	85.9	84.2	84.7	83.2	85.7	84,0	82,0	83.6	81.9	81.1	84.6	84.7	83.7	83.4	84.3	84.7	85.2
G4P6/Po/USA/Gottfried	87,0	82.7	86.4	85.3	86,0	81.9	86.6	81,0	82.2	81,0	82,0	80.9	87.1	86,0	82,0	85.9	85.9	86.6	82.3
G4P8/Hu/JPN/Hochi	85.3	84.2	86.2	84.7	85.3	83.6	86,0	83.9	81.7	83.4	81,0	81.1	85.2	85.3	84.2	84,0	84.6	84.4	84.4
G4P6/Po/THA/CMP77	91.3	82.2	91.3	91.1	92.6	81.5	91.8	82.2	81.6	81.5	80.7	81.8	93.8	92.6	81.9	91.4	92.3	91.7	81.7
G4P6/Hu/GBR/ST3	85.3	84.6	86.4	84.7	85.6	83.7	86.3	84.3	81.9	83.9	81.2	81.5	85.4	85.6	84.6	84.3	84.9	84.4	84.6
G4P8/Hu/POL/31	84.4	83.7	85.2	83.5	84.1	82.2	85.4	83.4	81.4	82.7	81.5	80.4	83.9	84.1	83.4	83.1	83.9	84.1	84.2
G4P8/Hu/POL/162	84.5	83.8	85.6	83.7	84.8	82.7	85.5	83.5	81.8	83.1	82,0	81.1	84.4	84.8	83.5	83.5	84.4	84.5	84.7
G4P6/Hu/POL/188	93.7	83.3	**98.1**	93.8	94.7	83,0	**99,0**	83.7	82.6	83.4	83.2	83.9	93.3	94.7	83.4	93.4	94.7	95.4	82.2
G4P8/Hu/POL/315	84.4	83.7	85.5	83.7	84.7	82.5	85.4	83.4	81.7	83.2	81.8	81,0	84.2	84.7	83.5	83.4	84.2	84.4	84.5
G4P8/Hu/POL/316	84.2	83.5	85.4	83.5	84.5	82.4	85.2	83.2	81.5	83.1	81.7	80.9	84.2	84.5	83.4	83.4	84.1	84.2	84.4
G4P8/Hu/POL/335	84.2	83.8	85.4	83.7	84.5	82.7	85.2	83.5	81.8	83.1	82,0	81.1	84.1	84.5	83.2	83.2	84.1	84.2	84.7
G4P8/Hu/POL/337	84.4	83.9	85.5	83.8	84.7	82.8	85.4	83.7	82,0	83.2	82.1	81.3	84.2	84.7	83.4	83.4	84.2	84.4	84.8
G4P8/Hu/POL/343	84.4	83.9	85.5	83.8	84.7	82.8	85.4	83.7	82,0	83.2	82.1	81.3	84.2	84.7	83.4	83.4	84.2	84.2	84.8
G4P8/Hu/POL/345	84.2	83.8	85.4	83.7	84.5	82.7	85.2	83.5	81.8	83.1	82.2	81.1	84.1	84.5	83.2	83.2	84.1	84.2	84.7
G4P8/Hu/POL/347	84.2	83.8	85.4	83.7	84.5	82.7	85.5	83.5	81.8	83.1	82,0	81.1	84.1	84.5	83.2	83.2	84.4	84.2	84.7
G4P8/Hu/POL/355	84.5	83.8	85.6	83.9	84.8	82.7	85.5	83.5	82.1	83.1	82.2	81.4	84.4	84.8	83.2	83.5	84.4	84.5	84.7
G4P6/Po/CHE/S19-1115	90.1	83.4	90.8	89.3	91.3	82.7	91.7	82.2	82.6	82,0	83,0	82.6	89.7	91.3	82.6	90.6	91.1	90.8	82.9
G4P6/Po/HRV/S243-VS	84,0	86,0	83,0	83.2	83.7	87.4	83.3	87.7	85.6	87,0	86.7	87.5	82.2	83.7	86,0	81.5	83.4	83.2	87.9
G4P6/Po/SVK/TOPC28	83.6	80.9	84.9	83.2	85.7	80.7	84.7	81,0	81.5	80.7	81.7	82.2	84.3	85.7	80,0	84.7	85.3	84,0	81.6
G4P6/Po/SVK/LSKC30	86.2	81.7	86,0	85,0	86.7	83.3	85.9	82.7	83.2	82.3	83.3	83.5	85.6	86.7	82.6	85.7	86.3	86.3	83,0
G4P6/Po/SVK/LSKC25	86.2	81.7	86,0	85,0	86.7	83.3	85.9	82.7	83.2	82.3	83.3	83.5	85.6	86.7	82.6	85.7	86.3	86.3	83,0
G4P6/Po/SVK/LSKC24	86.2	81.7	86,0	85,0	86.7	83.3	85.9	82.7	83.2	82.3	83.3	83.5	85.6	86.7	82.6	85.7	86.3	86.3	83,0
G4P6/Hu/ITA/PZ3	82.6	84.6	82.7	82.7	82.7	84,0	83.2	85.3	**83.4**	84.7	**83.4**	83.6	82,0	82.7	84.6	82.3	82.3	82.3	83.7
G4P6/Po/HRV/S400-VS	81,0	84.6	82.2	81.7	83.4	84.2	82.5	84.9	91.1	85.6	91.1	85.9	82,0	83.4	84.3	83.2	83.6	82.6	86.2
G4P6/Hu/HUN/BP1227	80.7	84.9	82.2	81.7	83.2	85.3	82.2	84.6	91.7	85.9	91.4	85.6	82.2	83.2	84.6	83.2	83.3	81.7	86.2

**RVA strain**	G4P6/Po/POL/597	G4P6/Po/POL/825	G4P6/Po/POL/921	G4P6/Po/POL/923	G4P6/Po/POL/1373	G4P6/Po/POL/871	G4P6/Po/POL/1421	G4P6/Po/POL/874	G4P6/Po/POL/1225	G4P6/Po/THA/CMP070	G4P8/Hu/GRC/Ath113	G4P8/Hu/BEL/BE1129	G4P8/Hu/USA/Bethesda	G4P8/Hu/DEU/GER124-09	G4P6/Po/USA/Gottfried	G4P8/Hu/JPN/Hochi	G4P6/Po/THA/CMP77
G4P6/Po/POL/962	84.3	93.1	93.1	93.1	92.3	93.4	82.9	84.3	83.4	93.4	84.9	85.2	85.4	85.3	87,0	85.3	91.3
G4P6/Po/POL/53	82.3	83.2	83.2	83.2	81.6	83.6	90.8	82.3	93.4	82.7	84.2	83.9	84.2	84,0	82.7	84.2	82.2
G4P6/Po/POL/1046	85.3	92.8	92.8	92.8	92.7	94.4	82.3	85.3	83.2	93.7	85.7	85.7	86.6	85.9	86.4	86.2	91.3
G4P6/Po/POL/920	84.4	99.7	99.7	99.7	92,0	93,0	81.6	84.4	83.6	92.3	84,0	84,0	84.9	84.2	85.3	84.7	91.1
G4P6/Po/POL/616	85.4	92.7	92.7	92.7	94.4	94.1	82.5	85.4	82.6	93.8	84.7	84.6	85.7	84.7	86,0	85.3	92.6
G4P6/Po/POL/1194	82.5	83.3	83.3	83.3	81.3	82.5	93.5	82.5	91.4	82,0	83,0	83,0	84.2	83.2	81.9	83.6	81.5
G4P6/Po/POL/868	85.7	94,0	94,0	94,0	93.5	95.3	82,0	85.7	83.2	94.5	85.6	85.6	86.7	85.7	86.6	86,0	91.8
G4P6/Po/POL/1224	82,0	83.6	83.6	83.6	81,0	83.4	91.7	82,0	100,0	82.2	84.2	84.2	83.9	84,0	81,0	83.9	82.2
G4P6/Po/POL/786	81.7	81.7	81.7	81.7	82.3	82.3	86.3	81.7	83.9	82.5	82.2	81.9	82.2	82,0	82.2	81.7	81.6
G4P6/Po/POL/1099	81.6	83.2	83.2	83.2	80.9	83,0	91.7	81.6	95.8	81.7	83.4	83.4	83.7	83.6	81,0	83.4	81.5
G4P6/Po/POL/37	82.3	81.7	81.7	81.7	82.5	83,0	86.4	82.3	83.9	81.9	81.9	81.7	81.7	81.9	82,0	81,0	80.7
G4P6/Po/POL/139	82.8	82.5	82.5	82.5	82.1	83.5	87.3	82.8	87.7	82.3	81.2	81.2	81.6	81.1	80.9	81.1	81.8
G4P6/Po/POL/1306	85,0	91.6	91.6	91.6	92.1	92.4	81.9	85,0	82.3	95.1	84.4	84.4	85.3	84.6	87.1	85.2	93.8
G4P6/Po/POL/1185	85.4	92.7	92.7	92.7	94.4	94.1	82.5	85.4	82.6	93.8	84.7	84.6	85.7	84.7	86,0	85.3	92.6
G4P6/Po/POL/964	82,0	83,0	83,0	83,0	81.6	82.9	90.7	82,0	93,0	82.3	83.9	83.6	84.2	83.7	82,0	84.2	81.9
G4P6/Po/POL/1372	84.7	92,0	92,0	92,0	100,0	93.7	81.5	84.7	81,0	93.4	83.3	83.3	84.4	83.4	85.9	84,0	91.4
G4P6/Po/POL/822	85.4	93.3	93.3	93.3	94.3	94.1	82.2	85.4	82.7	94,0	84.4	84.3	85,0	84.3	85.9	84.6	92.3
G4P6/Po/POL/870	84.7	93,0	93,0	93,0	93.7	100,0	82.3	84.7	83.4	94.1	84.3	84.9	84.6	84.7	86.6	84.4	91.7
G4P6/Po/POL/790	82.3	81.5	81.5	81.5	81.3	82.2	99.8	82.3	91.6	81.6	85,0	85,0	84.7	85.2	82.3	84.4	81.7
G4P6/Po/POL/597	-	84.4	84.4	84.4	84.7	84.7	82.5	100,0	82,0	84,0	88,0	88.9	89.7	88.7	86,0	89.1	85,0
G4P6/Po/POL/825	84.4	-	100,0	100,0	92,0	93,0	81.6	84.4	83.6	92.3	84,0	84,0	84.9	84.2	85.3	84.7	91.1
G4P6/Po/POL/921	84.4	100,0	-	100,0	92,0	93,0	81.6	84.4	83.6	92.3	84,0	84,0	84.9	84.2	85.3	84.7	91.1
G4P6/Po/POL/923	84.4	100,0	100,0	-	92,0	93,0	81.6	84.4	83.6	92.3	84,0	84,0	84.9	84.2	85.3	84.7	91.1
G4P6/Po/POL/1373	84.7	92,0	92,0	92,0	-	93.7	81.5	84.7	81,0	93.4	83.3	83.3	84.4	83.4	85.9	84,0	91.4
G4P6/Po/POL/871	84.7	93,0	93,0	93,0	93.7	-	82.3	84.7	83.4	94.1	84.3	84.9	84.6	84.7	86.6	84.4	91.7
G4P6/Po/POL/1421	82.5	81.6	81.6	81.6	81.5	82.3	-	82.5	91.7	81.7	85.2	85.2	84.9	85.3	82.5	84.6	81.9
G4P6/Po/POL/874	100,0	84.4	84.4	84.4	84.7	84.7	82.5	-	82,0	84,0	88,0	88.9	89.7	88.7	86,0	89.1	85,0
G4P6/Po/POL/1225	82,0	83.6	83.6	83.6	81,0	83.4	91.7	82,0	-	82.2	84.2	84.2	83.9	84,0	81,0	83.9	82.2
G4P6/Po/THA/CMP070	84,0	92.3	92.3	92.3	93.4	94.1	81.7	84,0	82.2	-	84.6	84.6	85.4	84.7	86.3	85.3	94.1
G4P8/Hu/GRC/Ath113	88,0	84,0	84,0	84,0	83.3	84.3	85.2	88,0	84.2	84.6	-	99.1	96.1	99.2	87.1	96.3	84.3
G4P8/Hu/BEL/BE1129	88.9	84,0	84,0	84,0	83.3	84.9	85.2	88.9	84.2	84.6	99.1	-	96.7	99.8	87.1	96.8	84.3
G4P8/Hu/USA/Bethesda	89.7	84.9	84.9	84.9	84.4	84.6	84.9	89.7	83.9	85.4	96.1	96.7	-	96.8	86.3	98.2	84.7
G4P8/Hu/DEU/GER124-09	88.7	84.2	84.2	84.2	83.4	84.7	85.3	88.7	84,0	84.7	99.2	99.8	96.8	-	87.3	97,0	84.2
G4P6/Po/USA/Gottfried	86,0	85.3	85.3	85.3	85.9	86.6	82.5	86,0	81,0	86.3	87.1	87.1	86.3	87.3	-	86.4	85,0
G4P8/Hu/JPN/Hochi	89.1	84.7	84.7	84.7	84,0	84.4	84.6	89.1	83.9	85.3	96.3	96.8	98.2	97,0	86.4	-	84.3
G4P6/Po/THA/CMP77	85,0	91.1	91.1	91.1	91.4	91.7	81.9	85,0	82.2	94.1	84.3	84.3	84.7	84.2	85,0	84.3	-
G4P6/Hu/GBR/ST3	88.7	85,0	85,0	85,0	84.3	84.4	84.7	88.7	84.3	85.9	96.1	96.7	98.1	96.8	86.3	98.4	84.6
G4P8/Hu/POL/31	88.1	83.5	83.5	83.5	83.1	84.1	84.4	88.1	83.4	84.1	98.8	98.3	95.8	98.4	86.4	95.7	83.5
G4P8/Hu/POL/162	87.6	83.7	83.7	83.7	83.5	84.5	84.8	87.6	83.5	84.8	98.7	98.4	95.4	98.5	86.8	95.6	83.9
G4P6/Hu/POL/188	85.6	93.8	93.8	93.8	93.4	95.4	82.3	85.6	83.7	94.7	85.4	85.4	86.3	85.6	86.4	85.9	91.7
G4P8/Hu/POL/315	87.5	83.7	83.7	83.7	83.4	84.4	84.7	87.5	83.4	84.7	98.5	98.3	95.3	98.4	86.6	95.4	83.9
G4P8/Hu/POL/316	87.5	83.5	83.5	83.5	83.4	84.2	84.5	87.5	83.2	84.5	98.4	98.1	95.1	98.3	86.5	95.3	83.9
G4P8/Hu/POL/335	87.3	83.7	83.7	83.7	83.2	84.2	84.8	87.3	83.5	84.5	98.4	98.1	95.1	98.3	86.5	95.3	83.9
G4P8/Hu/POL/337	87.5	83.8	83.8	83.8	83.4	84.4	84.9	87.5	83.7	84.7	98.5	98.3	95.3	98.4	86.6	95.4	84.1
G4P8/Hu/POL/343	87.5	83.8	83.8	83.8	83.4	84.2	84.9	87.5	83.7	84.7	98.4	98.1	95.3	98.3	86.6	95.4	83.9
G4P8/Hu/POL/345	87.3	83.7	83.7	83.7	83.2	84.2	84.8	87.3	83.5	84.5	98.4	98.1	95.1	98.3	86.8	95.3	83.9
G4P8/Hu/POL/347	87.6	83.7	83.7	83.7	83.2	84.2	84.8	87.6	83.5	84.5	98.4	98.1	95.4	98.3	86.5	95.3	83.9
G4P8/Hu/POL/355	87.6	83.9	83.9	83.9	83.5	84.5	84.8	87.6	83.5	84.8	98.4	98.1	95.4	98.3	86.5	95.3	84.2
G4P6/Po/CHE/S19-1115	85.6	89.3	89.3	89.3	90.6	90.8	83,0	85.6	82.2	90,0	84.9	85.2	85.2	85,0	86.3	85,0	89.3
G4P6/Po/HRV/S243-VS	83,0	82.9	82.9	82.9	81.5	83.2	88,0	83,0	87.7	82.7	82.7	83,0	83,0	82.9	81.7	83,0	81.9
G4P6/Po/SVK/TOPC28	92.8	82.9	82.9	82.9	84.7	84,0	81.7	92.8	81,0	84.3	87.9	88.4	89.3	88.3	85.4	88.4	84.7
G4P6/Po/SVK/LSKC30	86.9	85,0	85,0	85,0	85.7	86.3	83.2	86.9	82.7	85,0	86.2	86.7	86.7	86.6	86,0	87,0	85.9
G4P6/Po/SVK/LSKC25	86.9	85,0	85,0	85,0	85.7	86.3	83.2	86.9	82.7	85,0	86.2	86.7	86.7	86.6	86,0	87,0	85.9
G4P6/Po/SVK/LSKC24	86.9	85,0	85,0	85,0	85.7	86.3	83.2	86.9	82.7	85,0	86.2	86.7	86.7	86.6	86,0	87,0	85.9
G4P6/Hu/ITA/PZ3	82.5	82.7	82.7	82.7	82.3	82.3	83.9	82.5	85.3	82.3	82,0	82,0	82.5	82.2	81.6	82.3	81.9
G4P6/Po/HRV/S400-VS	82.7	81.7	81.7	81.7	83.2	82.6	86.3	82.7	84.9	81.7	82.9	82.7	82.9	82.9	83,0	82.7	81.5
G4P6/Hu/HUN/BP1227	82.2	81.7	81.7	81.7	83.2	81.7	86.3	82.2	84.6	81.6	82.5	82,0	82.2	82.2	82.5	82,0	81.2

RVA strain	G4P6/Hu/GBR/ST3	G4P8/Hu/POL/31	G4P8/Hu/POL/162	G4P6/Hu/POL/188	G4P8/Hu/POL/315	G4P8/Hu/POL/316	G4P8/Hu/POL/335	G4P8/Hu/POL/337	G4P8/Hu/POL/343	G4P8/Hu/POL/345	G4P8/Hu/POL/347	G4P8/Hu/POL/355	G4P6/Po/CHE/S19-1115	G4P6/Po/HRV/S243-VS	G4P6/Po/SVK/TOPC28	G4P6/Po/SVK/LSKC30	G4P6/Po/SVK/LSKC25	G4P6/Po/SVK/LSKC24
G4P6/Po/POL/962	85.3	84.4	84.5	93.7	84.4	84.2	84.2	84.4	84.4	84.2	84.2	84.5	90.1	84,0	83.6	86.2	86.2	86.2
G4P6/Po/POL/53	84.6	83.7	83.8	83.3	83.7	83.5	83.8	83.9	83.9	83.8	83.8	83.8	83.4	86,0	80.9	81.7	81.7	81.7
G4P6/Po/POL/1046	86.4	85.2	85.6	98.1	85.5	85.4	85.4	85.5	85.5	85.4	85.4	85.6	90.8	83,0	84.9	86,0	86,0	86,0
G4P6/Po/POL/920	84.7	83.5	83.7	93.8	83.7	83.5	83.7	83.8	83.8	83.7	83.7	83.9	89.3	83.2	83.2	85,0	85,0	85,0
G4P6/Po/POL/616	85.6	84.1	84.8	94.7	84.7	84.5	84.5	84.7	84.7	84.5	84.5	84.8	91.3	83.7	85.7	86.7	86.7	86.7
G4P6/Po/POL/1194	83.7	82.2	82.7	83,0	82.5	82.4	82.7	82.8	82.8	82.7	82.7	82.7	82.7	87.4	80.7	83.3	83.3	83.3
G4P6/Po/POL/868	86.3	85.4	85.5	99,0	85.4	85.2	85.2	85.4	85.4	85.2	85.5	85.5	91.7	83.3	84.7	85.9	85.9	85.9
G4P6/Po/POL/1224	84.3	83.4	83.5	83.7	83.4	83.2	83.5	83.7	83.7	83.5	83.5	83.5	82.2	87.7	81,0	82.7	82.7	82.7
G4P6/Po/POL/786	81.9	81.4	81.8	82.6	81.7	81.5	81.8	82,0	82,0	81.8	81.8	82.1	82.6	85.6	81.5	83.2	83.2	83.2
G4P6/Po/POL/1099	83.9	82.7	83.1	83.4	83.2	83.1	83.1	83.2	83.2	83.1	83.1	83.1	82,0	87,0	80.7	82.3	82.3	82.3
G4P6/Po/POL/37	81.2	81.5	82,0	83.2	81.8	81.7	82,0	82.1	82.1	82.2	82,0	82.2	83,0	86.7	81.7	83.3	83.3	83.3
G4P6/Po/POL/139	81.5	80.4	81.1	83.9	81,0	80.9	81.1	81.3	81.3	81.1	81.1	81.4	82.6	87.5	82.2	83.5	83.5	83.5
G4P6/Po/POL/1306	85.4	83.9	84.4	93.3	84.2	84.2	84.1	84.2	84.2	84.1	84.1	84.4	89.7	82.2	84.3	85.6	85.6	85.6
G4P6/Po/POL/1185	85.6	84.1	84.8	94.7	84.7	84.5	84.5	84.7	84.7	84.5	84.5	84.8	91.3	83.7	85.7	86.7	86.7	86.7
G4P6/Po/POL/964	84.6	83.4	83.5	83.4	83.5	83.4	83.2	83.4	83.4	83.2	83.2	83.2	82.6	86,0	80,0	82.6	82.6	82.6
G4P6/Po/POL/1372	84.3	83.1	83.5	93.4	83.4	83.4	83.2	83.4	83.4	83.2	83.2	83.5	90.6	81.5	84.7	85.7	85.7	85.7
G4P6/Po/POL/822	84.9	83.9	84.4	94.7	84.2	84.1	84.1	84.2	84.2	84.1	84.4	84.4	91.1	83.4	85.3	86.3	86.3	86.3
G4P6/Po/POL/870	84.4	84.1	84.5	95.4	84.4	84.2	84.2	84.4	84.2	84.2	84.2	84.5	90.8	83.2	84,0	86.3	86.3	86.3
G4P6/Po/POL/790	84.6	84.2	84.7	82.2	84.5	84.4	84.7	84.8	84.8	84.7	84.7	84.7	82.9	87.9	81.6	83,0	83,0	83,0
G4P6/Po/POL/597	88.7	88.1	87.6	85.6	87.5	87.5	87.3	87.5	87.5	87.3	87.6	87.6	85.6	83,0	92.8	86.9	86.9	86.9
G4P6/Po/POL/825	85,0	83.5	83.7	93.8	83.7	83.5	83.7	83.8	83.8	83.7	83.7	83.9	89.3	82.9	82.9	85,0	85,0	85,0
G4P6/Po/POL/921	85,0	83.5	83.7	93.8	83.7	83.5	83.7	83.8	83.8	83.7	83.7	83.9	89.3	82.9	82.9	85,0	85,0	85,0
G4P6/Po/POL/923	85,0	83.5	83.7	93.8	83.7	83.5	83.7	83.8	83.8	83.7	83.7	83.9	89.3	82.9	82.9	85,0	85,0	85,0
G4P6/Po/POL/1373	84.3	83.1	83.5	93.4	83.4	83.4	83.2	83.4	83.4	83.2	83.2	83.5	90.6	81.5	84.7	85.7	85.7	85.7
G4P6/Po/POL/871	84.4	84.1	84.5	95.4	84.4	84.2	84.2	84.4	84.2	84.2	84.2	84.5	90.8	83.2	84,0	86.3	86.3	86.3
G4P6/Po/POL/1421	84.7	84.4	84.8	82.3	84.7	84.5	84.8	84.9	84.9	84.8	84.8	84.8	83,0	88,0	81.7	83.2	83.2	83.2
G4P6/Po/POL/874	88.7	88.1	87.6	85.6	87.5	87.5	87.3	87.5	87.5	87.3	87.6	87.6	85.6	83,0	92.8	86.9	86.9	86.9
G4P6/Po/POL/1225	84.3	83.4	83.5	83.7	83.4	83.2	83.5	83.7	83.7	83.5	83.5	83.5	82.2	87.7	81,0	82.7	82.7	82.7
G4P6/Po/THA/CMP070	85.9	84.1	84.8	94.7	84.7	84.5	84.5	84.7	84.7	84.5	84.5	84.8	90,0	82.7	84.3	85,0	85,0	85,0
G4P8/Hu/GRC/Ath113	96.1	98.8	98.7	85.4	98.5	98.4	98.4	98.5	98.4	98.4	98.4	98.4	84.9	82.7	87.9	86.2	86.2	86.2
G4P8/Hu/BEL/BE1129	96.7	98.3	98.4	85.4	98.3	98.1	98.1	98.3	98.1	98.1	98.1	98.1	85.2	83,0	88.4	86.7	86.7	86.7
G4P8/Hu/USA/Bethesda	98.1	95.8	95.4	86.3	95.3	95.1	95.1	95.3	95.3	95.1	95.4	95.4	85.2	83,0	89.3	86.7	86.7	86.7
G4P8/Hu/DEU/GER124-09	96.8	98.4	98.5	85.6	98.4	98.3	98.3	98.4	98.3	98.3	98.3	98.3	85,0	82.9	88.3	86.6	86.6	86.6
G4P6/Po/USA/Gottfried	86.3	86.4	86.8	86.4	86.6	86.5	86.5	86.6	86.6	86.8	86.5	86.5	86.3	81.7	85.4	86,0	86,0	86,0
G4P8/Hu/JPN/Hochi	98.4	95.7	95.6	85.9	95.4	95.3	95.3	95.4	95.4	95.3	95.3	95.3	85,0	83,0	88.4	87,0	87,0	87,0
G4P6/Po/THA/CMP77	84.6	83.5	83.9	91.7	83.9	83.9	83.9	84.1	83.9	83.9	83.9	84.2	89.3	81.9	84.7	85.9	85.9	85.9
G4P6/Hu/GBR/ST3	-	95.6	95.4	86.2	95.3	95.1	95.1	95.3	95.3	95.1	95.1	95.1	84.3	82.6	88.6	86.9	86.9	86.9
G4P8/Hu/POL/31	95.6	-	98.8	84.9	98.7	98.5	98.5	98.7	98.5	98.5	98.8	98.5	84.7	82,0	87.9	85.1	85.1	85.1
G4P8/Hu/POL/162	95.4	98.8	-	85.4	99.8	99.7	99.7	99.8	99.7	99.7	99.7	99.7	85.1	82.4	88.1	85.5	85.5	85.5
G4P6/Hu/POL/188	86.2	**84.9**	**85.4**	**-**	**85.2**	**85.1**	**85.1**	**85.2**	**85.2**	**85.1**	**85.1**	**85.4**	91.3	84,0	84.3	86,0	86,0	86,0
G4P8/Hu/POL/315	95.3	98.7	99.8	85.2	-	99.8	99.5	99.7	99.5	99.5	99.5	99.5	84.9	82.2	87.9	85.4	85.4	85.4
G4P8/Hu/POL/316	95.1	98.5	99.7	85.1	99.8	-	99.4	99.5	99.4	99.4	99.4	99.4	84.8	82.1	87.9	85.2	85.2	85.2
G4P8/Hu/POL/335	95.1	98.5	99.7	85.1	99.5	99.4	-	99.8	99.7	99.7	99.7	99.7	84.8	82.4	88.1	85.2	85.2	85.2
G4P8/Hu/POL/337	95.3	98.7	99.8	85.2	99.7	99.5	99.8	-	99.8	99.8	99.8	99.8	84.9	82.5	88.2	85.4	85.4	85.4
G4P8/Hu/POL/343	95.3	98.5	99.7	85.2	99.5	99.4	99.7	99.8	-	99.7	99.7	99.7	84.9	82.5	88.2	85.4	85.4	85.4
G4P8/Hu/POL/345	95.1	98.5	99.7	85.1	99.5	99.4	99.7	99.8	99.7	-	99.7	99.7	85.1	82.4	88.1	85.5	85.5	85.5
G4P8/Hu/POL/347	95.1	98.8	99.7	85.1	99.5	99.4	99.7	99.8	99.7	99.7	-	99.7	85.1	82.4	88.3	85.2	85.2	85.2
G4P8/Hu/POL/355	95.1	98.5	99.7	85.4	99.5	99.4	99.7	99.8	99.7	99.7	99.7	-	85.1	82.7	88.3	85.2	85.2	85.2
G4P6/Po/CHE/S19-1115	84.3	84.7	85.1	91.3	84.9	84.8	84.8	84.9	84.9	85.1	85.1	85.1	-	84.2	85.3	85.6	85.6	85.6
G4P6/Po/HRV/S243-VS	82.6	82,0	82.4	84,0	82.2	82.1	82.4	82.5	82.5	82.4	82.4	82.7	84.2	-	82.6	82.5	82.5	82.5
G4P6/Po/SVK/TOPC28	88.6	87.9	88.1	84.3	87.9	87.9	88.1	88.2	88.2	88.1	88.3	88.3	85.3	82.6	-	85.4	85.4	85.4
G4P6/Po/SVK/LSKC30	86.9	85.1	85.5	86,0	85.4	85.2	85.2	85.4	85.4	85.5	85.2	85.2	85.6	82.5	85.4	-	100,0	100,0
G4P6/Po/SVK/LSKC25	86.9	85.1	85.5	86,0	85.4	85.2	85.2	85.4	85.4	85.5	85.2	85.2	85.6	82.5	85.4	100,0	-	100,0
G4P6/Po/SVK/LSKC24	86.9	85.1	85.5	86,0	85.4	85.2	85.2	85.4	85.4	85.5	85.2	85.2	85.6	82.5	85.4	100,0	100,0	-
G4P6/Hu/ITA/PZ3	82.3	81.5	82,0	83.3	81.8	81.7	82,0	82.1	82.1	82.2	82,0	82.2	82.3	83.9	81.6	82.5	82.5	82.5
G4P6/Po/HRV/S400-VS	82.9	82.8	83,0	82.7	82.8	82.7	83,0	83.1	83.1	83,0	83,0	83.2	82.7	86.2	83,0	83.3	83.3	83.3
G4P6/Hu/HUN/BP1227	82.2	82.1	82.5	82.5	82.4	82.2	82.5	82.7	82.7	82.5	82.5	82.8	83.3	86.7	82.9	83,0	83,0	83,0

RVA strain	G4P6/Hu/ITA/PZ3	G4P6/Po/HRV/S400-VS	G4P6/Hu/HUN/BP1227
G4P6/Po/POL/962	82.6	81,0	80.7
G4P6/Po/POL/53	84.6	84.6	84.9
G4P6/Po/POL/1046	82.7	82.2	82.2
G4P6/Po/POL/920	82.7	81.7	81.7
G4P6/Po/POL/616	82.7	83.4	83.2
G4P6/Po/POL/1194	84,0	84.2	85.3
G4P6/Po/POL/868	83.2	82.5	82.2
G4P6/Po/POL/1224	85.3	84.9	84.6
G4P6/Po/POL/786	83.4	91.1	91.7
G4P6/Po/POL/1099	84.7	85.6	85.9
G4P6/Po/POL/37	83.4	91.1	91.4
G4P6/Po/POL/139	83.6	85.9	85.6
G4P6/Po/POL/1306	82,0	82,0	82.2
G4P6/Po/POL/1185	82.7	83.4	83.2
G4P6/Po/POL/964	84.6	84.3	84.6
G4P6/Po/POL/1372	82.3	83.2	83.2
G4P6/Po/POL/822	82.3	83.6	83.3
G4P6/Po/POL/870	82.3	82.6	81.7
G4P6/Po/POL/790	83.7	86.2	86.2
G4P6/Po/POL/597	82.5	82.7	82.2
G4P6/Po/POL/825	82.7	81.7	81.7
G4P6/Po/POL/921	82.7	81.7	81.7
G4P6/Po/POL/923	82.7	81.7	81.7
G4P6/Po/POL/1373	82.3	83.2	83.2
G4P6/Po/POL/871	82.3	82.6	81.7
G4P6/Po/POL/1421	83.9	86.3	86.3
G4P6/Po/POL/874	82.5	82.7	82.2
G4P6/Po/POL/1225	85.3	84.9	84.6
G4P6/Po/THA/CMP070	82.3	81.7	81.6
G4P8/Hu/GRC/Ath113	82,0	82.9	82.5
G4P8/Hu/BEL/BE1129	82,0	82.7	82,0
G4P8/Hu/USA/Bethesda	82.5	82.9	82.2
G4P8/Hu/DEU/GER124-09	82.2	82.9	82.2
G4P6/Po/USA/Gottfried	81.6	83,0	82.5
G4P8/Hu/JPN/Hochi	82.3	82.7	82,0
G4P6/Po/THA/CMP77	81.9	81.5	81.2
G4P6/Hu/GBR/ST3	82.3	82.9	82.2
G4P8/Hu/POL/31	81.5	82.8	82.1
G4P8/Hu/POL/162	82,0	83,0	82.5
G4P6/Hu/POL/188	83.3	82.7	82.5
G4P8/Hu/POL/315	81.8	82.8	82.4
G4P8/Hu/POL/316	81.7	82.7	82.2
G4P8/Hu/POL/335	82,0	83,0	82.5
G4P8/Hu/POL/337	82.1	83.1	82.7
G4P8/Hu/POL/343	82.1	83.1	82.7
G4P8/Hu/POL/345	82.2	83,0	82.5
G4P8/Hu/POL/347	82,0	83,0	82.5
G4P8/Hu/POL/355	82.2	83.2	82.8
G4P6/Po/CHE/S19-1115	82.3	82.7	83.3
G4P6/Po/HRV/S243-VS	83.9	86.2	86.7
G4P6/Po/SVK/TOPC28	81.6	83,0	82.9
G4P6/Po/SVK/LSKC30	82.5	83.3	83,0
G4P6/Po/SVK/LSKC25	82.5	83.3	83,0
G4P6/Po/SVK/LSKC24	82.5	83.3	83,0
G4P6/Hu/ITA/PZ3	-	83.2	83.2
G4P6/Po/HRV/S400-VS	83.2	-	95.7
G4P6/Hu/HUN/BP1227	83.2	95.7	-
