# Supplementary material for: Detection of Porcine–Human Reassortant and Zoonotic Group A Rotaviruses in Humans in Poland
Source: Transbound Emerg Dis. 2024 Sep 24;2024:4232389. doi: 10.1155/2024/4232389 (PMC12017087; doi:10.1155/2024/4232389)
Supplement: Supporting Information S4 — Table 4: the nucleotide sequence similarity of the VP7 gene fragment of pig and human G9 RVA strains. [file 4232389.f4.pdf]

Supplementary Table S4. The nucleotide sequence similarity of the VP7 gene fragment of pig and human G9 RVA strains

**RVA strain**	G9P6/Po/POL/38	G9P6/Po/POL/1307	G9P8/Hu/POL/82	G9P8/Hu/POL/49	G9P8/Hu/POL/95	G9P8/Hu/POL/150	G9P8/Hu/POL/38	G9P8/Hu/POL/219	G9P8/Hu/POL/180	G9P8/Hu/POL/104	G9P8/Hu/POL/109	G9P6/Po/POL/775	G9P6/Po/POL/1040	G9P6/Hu/BEL/BE1248	G9P6/Po/CAN/CE-M-05-0067	G9P8/Hu/USA/WI61
G9P6/Po/POL/38	-	0.965	0.914	0.913	0.913	0.914	0.911	0.909	0.911	0.911	0.935	0.954	0.951	0.917	0.913	0.879
G9P6/Po/POL/1307	0.965	-	0.917	0.915	0.915	0.917	0.911	0.911	0.914	0.911	0.937	0.943	0.941	0.911	0.911	0.879
G9P8/Hu/POL/82	0.914	0.917	-	0.998	0.998	1	0.981	0.994	0.997	0.981	0.921	0.899	0.897	0.918	0.913	0.893
G9P8/Hu/POL/49	0.913	0.915	0.998	-	0.997	0.998	0.979	0.995	0.998	0.979	0.919	0.901	0.898	0.919	0.911	0.891
G9P8/Hu/POL/95	0.913	0.915	0.998	0.997	-	0.998	0.979	0.993	0.995	0.979	0.919	0.898	0.895	0.917	0.911	0.891
G9P8/Hu/POL/150	0.914	0.917	1	0.998	0.998	-	0.981	0.994	0.997	0.981	0.921	0.899	0.897	0.918	0.913	0.893
G9P8/Hu/POL/38	0.911	0.911	0.981	0.979	0.979	0.981	-	0.975	0.978	0.997	0.918	0.897	0.893	0.921	0.91	0.89
G9P8/Hu/POL/219	0.909	0.911	0.994	0.995	0.993	0.994	0.975	-	0.997	0.975	0.915	0.897	0.894	0.915	0.907	0.887
G9P8/Hu/POL/180	0.911	0.914	0.997	0.998	0.995	0.997	0.978	0.997	-	0.978	0.918	0.899	0.897	0.918	0.91	0.89
G9P8/Hu/POL/104	0.911	0.911	0.981	0.979	0.979	0.981	0.997	0.975	0.978	-	0.918	0.895	0.891	0.921	0.91	0.89
G9P8/Hu/POL/109	0.935	0.937	0.921	0.919	0.919	0.921	0.918	0.915	0.918	0.918	-	0.918	0.915	0.921	0.913	0.882
G9P6/Po/POL/775	0.954	0.943	0.899	0.901	0.898	0.899	0.897	0.897	0.899	0.895	0.918	-	0.982	0.899	0.894	0.863
G9P6/Po/POL/1040	0.951	0.941	0.897	0.898	0.895	0.897	0.893	0.894	0.897	0.891	0.915	0.982	-	0.895	0.89	0.857
G9P6/Hu/BEL/BE1248	0.917	0.911	0.918	0.919	0.917	0.918	0.921	0.915	0.918	0.921	0.921	0.899	0.895	-	0.901	0.881
G9P6/Po/CAN/CE-M-05-0067	0.913	0.911	0.913	0.911	0.911	0.913	0.91	0.907	0.91	0.91	0.913	0.894	0.89	0.901	-	0.886
G9P8/Hu/USA/WI61	0.879	0.879	0.893	0.891	0.891	0.893	0.89	0.887	0.89	0.89	0.882	0.863	0.857	0.881	0.886	-
