# Supplementary material for: Detection of Porcine–Human Reassortant and Zoonotic Group A Rotaviruses in Humans in Poland
Source: Transbound Emerg Dis. 2024 Sep 24;2024:4232389. doi: 10.1155/2024/4232389 (PMC12017087; doi:10.1155/2024/4232389)
Supplement: Supporting Information S5 — Table 5: the nucleotide sequence similarity of the VP4 gene fragment of pig and human P[6] RVA strains. [file 4232389.f5.pdf]

Supplementary Table S5. The nucleotide sequence similarity of the VP4 gene fragment of pig and human P[6] RVA strains

**RVA strain**	G4P6/Hu/GBR/ST3	G4P6/Po/POL/53	G4P6/Po/POL/1046	G5P6/Po/POL/1075	G5P6/Po/POL/449	G4P6/Po/POL/1224	G4P6/Po/POL/786	G4P6/Po/POL/962	G4P6/Po/POL/920	G4P6/Po/POL/868	G4P6/Po/POL/1099	G5P6/Po/POL/857	G9P6/Po/POL/775	G9P6/Po/POL/1040	G5P6/Po/POL/620	G9P6/Po/POL/38	G9P6/Po/POL/1307	G3P6/Po/POL/823	G4P6/Po/POL/964	G4P6/Po/POL/822
G4P6/Hu/GBR/ST3	-	78.2	80.5	77.2	88.8	82.6	81.3	82.3	82.1	81.3	82.3	81.3	83.4	82.1	84.4	78.2	88.8	81.8	82.6	81.8
G4P6/Po/POL/53	78.2	-	94	80.3	81.3	83.6	83.9	83.4	85.7	84.4	84.4	83.4	83.9	84.7	83.4	100	81.8	84.4	83.9	84.4
G4P6/Po/POL/1046	80.5	94	-	81.3	83.1	84.9	86.2	85.7	86.5	84.1	86.7	85.4	86.7	85.4	84.1	94	83.6	84.7	86.2	84.7
G5P6/Po/POL/1075	77.2	80.3	81.3	-	79.2	82.3	81.6	83.4	80.5	81.3	81.8	81.8	83.4	79.7	79.5	80.3	79.5	78.7	83.6	78.7
G5P6/Po/POL/449	88.8	81.3	83.1	79.2	-	83.1	82.6	83.4	84.9	83.6	83.6	82.3	83.9	85.4	84.7	81.3	99.2	83.4	83.6	83.4
G4P6/Po/POL/1224	82.6	83.6	84.9	82.3	83.1	-	93.5	94	85.7	83.9	94.8	94.3	94.5	86	84.1	83.6	83.6	85.7	94.5	85.7
G4P6/Po/POL/786	81.3	83.9	86.2	81.6	82.6	93.5	-	94.8	85.4	83.6	95	95	95.8	84.7	84.4	83.9	83.1	85.4	95.3	85.4
G4P6/Po/POL/962	82.3	83.4	85.7	83.4	83.4	94	94.8	-	85.4	83.9	95.5	95.5	96.6	84.9	85.2	83.4	83.9	84.7	99.4	84.7
G4P6/Po/POL/920	82.1	85.7	86.5	80.5	84.9	85.7	85.4	85.4	-	94.8	85.2	84.4	86.5	96.6	84.1	85.7	85.4	95.3	85.7	95.3
G4P6/Po/POL/868	81.3	84.4	84.1	81.3	83.6	83.9	83.6	83.9	94.8	-	83.9	83.1	84.7	94.5	83.6	84.4	83.6	93.7	84.1	93.7
G4P6/Po/POL/1099	82.3	84.4	86.7	81.8	83.6	94.8	95	95.5	85.2	83.9	-	95.8	96.6	84.9	85.2	84.4	84.1	85.7	96.1	85.7
G5P6/Po/POL/857	81.3	83.4	85.4	81.8	82.3	94.3	95	95.5	84.4	83.1	95.8	-	96.6	84.1	84.1	83.4	82.9	84.4	96.1	84.4
G9P6/Po/POL/775	83.4	83.9	86.7	83.4	83.9	94.5	95.8	96.6	86.5	84.7	96.6	96.6	-	85.7	86	83.9	84.4	86	97.1	86
G9P6/Po/POL/1040	82.1	84.7	85.4	79.7	85.4	86	84.7	84.9	96.6	94.5	84.9	84.1	85.7	-	83.9	84.7	86	95.5	85.2	95.5
G5P6/Po/POL/620	84.4	83.4	84.1	79.5	84.7	84.1	84.4	85.2	84.1	83.6	85.2	84.1	86	83.9	-	83.4	84.7	82.1	85.4	82.1
G9P6/Po/POL/38	78.2	100	94	80.3	81.3	83.6	83.9	83.4	85.7	84.4	84.4	83.4	83.9	84.7	83.4	-	81.8	84.4	83.9	84.4
G9P6/Po/POL/1307	88.8	81.8	83.6	79.5	99.2	83.6	83.1	83.9	85.4	83.6	84.1	82.9	84.4	86	84.7	81.8	-	83.9	84.1	83.9
G3P6/Po/POL/823	81.8	84.4	84.7	78.7	83.4	85.7	85.4	84.7	95.3	93.7	85.7	84.4	86	95.5	82.1	84.4	83.9	-	84.9	100
G4P6/Po/POL/964	82.6	83.9	86.2	83.6	83.6	94.5	95.3	99.4	85.7	84.1	96.1	96.1	97.1	85.2	85.4	83.9	84.1	84.9	-	84.9
G4P6/Po/POL/822	81.8	84.4	84.7	78.7	83.4	85.7	85.4	84.7	95.3	93.7	85.7	84.4	86	95.5	82.1	84.4	83.9	100	84.9	-
G3P6/Po/POL/551	81.8	84.4	84.7	78.7	83.4	85.7	85.4	84.7	95.3	93.7	85.7	84.4	86	95.5	82.1	84.4	83.9	100	84.9	100
G4P6/Po/POL/825	81.8	84.4	84.7	78.7	83.4	85.7	85.4	84.7	95.3	93.7	85.7	84.4	86	95.5	82.1	84.4	83.9	100	84.9	100
G4P6/Po/POL/1306	88.8	81.8	83.6	79.7	99.4	83.6	83.1	83.9	85.4	83.6	84.1	82.9	84.4	86	84.7	81.8	99.7	83.9	84.1	83.9
G4P6/Po/POL/1372	84.4	83.4	84.1	79.5	84.7	84.1	84.4	85.2	84.1	83.6	85.2	84.1	86	83.9	100	83.4	84.7	82.1	85.4	82.1
G4P6/Po/POL/1373	84.4	83.4	84.1	79.5	84.7	84.1	84.4	85.2	84.1	83.6	85.2	84.1	86	83.9	100	83.4	84.7	82.1	85.4	82.1
G4P6/Po/POL/921	82.3	86	86.7	80.5	85.2	86	85.2	85.7	99.7	95	85.4	84.7	86.7	96.8	84.4	86	85.7	95.5	86	95.5
G4P6/Po/POL/923	82.3	86	86.7	80.5	85.2	86	85.2	85.7	99.7	95	85.4	84.7	86.7	96.8	84.4	86	85.7	95.5	86	95.5
G4P6/Po/POL/1185	83.4	83.9	86.7	83.4	83.9	94.5	95.8	96.6	86.5	84.7	96.6	96.6	100	85.7	86	83.9	84.4	86	97.1	86
G4P6/Po/POL/1194	82.9	85.4	85.7	80.3	86.2	85.2	85.4	85.4	94.8	95.5	86.2	84.9	86.5	94.5	85.2	85.4	86.2	92.2	86	92.2
G4P6/Po/POL/874	82.9	86	86.7	79.7	85.2	86.2	86.5	85.2	96.3	94.3	86.2	85.4	87	96.6	83.6	86	85.7	96.3	85.4	96.3
G5P6/Po/POL/450	88.8	81.3	83.1	79.2	100	83.1	82.6	83.4	84.9	83.6	83.6	82.3	83.9	85.4	84.7	81.3	99.2	83.4	83.6	83.4
G4P6/Po/POL/597	83.6	82.1	83.4	78.4	83.4	83.9	83.1	84.9	82.6	82.1	84.9	84.4	85.7	82.3	96.8	82.1	83.4	81	85.2	81
G4P6/Po/POL/870	81.3	84.4	84.1	81.3	83.6	83.9	83.6	83.9	94.8	100	83.9	83.1	84.7	94.5	83.6	84.4	83.6	93.7	84.1	93.7
G4P6/Po/POL/871	81.3	84.4	84.1	81.3	83.6	83.9	83.6	83.9	94.8	100	83.9	83.1	84.7	94.5	83.6	84.4	83.6	93.7	84.1	93.7
G4P6/Po/POL/1225	82.6	83.6	84.9	82.3	83.1	100	93.5	94	85.7	83.9	94.8	94.3	94.5	86	84.1	83.6	83.6	85.7	94.5	85.7
G4P6/Po/POL/790	81.3	83.9	86.2	81.6	82.6	93.5	100	94.8	85.4	83.6	95	95	95.8	84.7	84.4	83.9	83.1	85.4	95.3	85.4
G4P6/Po/POL/139	90.4	81.8	83.6	78.7	91.4	85.2	85.7	84.7	84.7	83.1	86	84.9	86.5	84.9	84.9	81.8	91.4	84.9	85.2	84.9
G9P6/Po/POL/546	81.3	83.4	85.4	81.8	82.3	94.3	95	95.5	84.4	83.1	95.8	100	96.6	84.1	84.1	83.4	82.9	84.4	96.1	84.4
G5P6/Po/POL/733	83.4	83.9	86.7	83.4	83.9	94.5	95.8	96.6	86.5	84.7	96.6	96.6	100	85.7	86	83.9	84.4	86	97.1	86
G4P6/Hu/POL/188	76.1	79	77.9	**100**	77.9	81.3	82.6	81.3	78.2	78.7	81	80.8	82.1	79.2	80	79	78.2	77.7	81.6	77.7
G4P6/Po/CHE/S19-1116	79.7	85.4	86.2	82.1	82.3	84.1	83.9	85.7	91.1	89.8	84.7	84.4	85.7	90.6	83.9	85.4	82.9	89.3	86	89.3
G4P6/Po/CHE/S19-1115	79.7	85.4	86.2	82.1	82.3	84.1	83.9	85.7	91.1	89.8	84.7	84.4	85.7	90.6	83.9	85.4	82.9	89.3	86	89.3
G4P6/Po/HRV/S243-VS	82.6	85.4	86.2	81.6	84.9	86.2	87	87.3	92.2	91.7	86.7	87	88	92.4	84.9	85.4	85.4	90.6	87.5	90.6
G4P6/Po/SVK/TOPC28	76.9	79.5	80.5	96.6	79	82.1	82.3	84.1	79.7	80.5	82.1	82.1	83.6	79.5	77.7	79.5	79.2	79.2	84.4	79.2
G4P6/Po/SVK/LSKC30	82.6	84.4	85.7	80	85.2	85.4	86.2	86.5	93.5	92.2	86	85.7	87.3	93	85.4	84.4	85.7	91.7	86.7	91.7
G4P6/Po/SVK/LSKC25	82.3	84.7	86	80.3	84.9	85.7	86.5	86.7	93.7	92.4	86.2	86	87.5	92.7	85.7	84.7	85.4	91.9	87	91.9
G4P6/Po/SVK/LSKC24	82.3	84.7	86	80.3	84.9	85.7	86.5	86.7	93.7	92.4	86.2	86	87.5	92.7	85.7	84.7	85.4	91.9	87	91.9
G4P6/Hu/ITA/PZ3	89.3	81.3	83.1	80.3	93.2	84.1	83.1	84.9	83.9	82.6	84.1	82.9	83.4	84.4	87.5	81.3	93	82.6	85.2	82.6
G4P6/Po/HRV/S400-VS	83.4	84.9	84.4	82.3	83.9	85.7	84.9	84.7	83.6	85.2	86.2	84.7	85.4	83.4	87.3	84.9	83.6	82.6	84.9	82.6
G4P6/Po/HRV/S397-VS	83.4	84.9	84.4	82.3	83.9	85.7	84.9	84.7	83.6	85.2	86.2	84.7	85.4	83.4	87.3	84.9	83.6	82.6	84.9	82.6
G5P6/Po/SVN/P83	83.9	86.2	87	82.3	85.7	86.7	86.5	87.8	94.5	93	87.3	86.5	88.3	93.5	86.2	86.2	86.2	91.7	88	91.7
G4P6/Hu/HUN/BP1125	82.9	85.4	86.7	80.5	84.7	85.7	86.5	86.7	93.7	93	86.2	86	87	94	85.2	85.4	85.2	91.7	87	91.7
G4P6/Hu/HUN/BP1547	82.6	84.1	86.2	80	85.4	84.7	85.4	85.7	91.4	90.6	85.2	85.7	86	91.9	84.9	84.1	86	89.6	86	89.6
G5P6/Po/GBR/X1	82.6	82.3	83.1	80.8	83.4	86	85.4	86	83.4	84.1	86	85.4	86.5	83.6	85.2	82.3	83.4	81.8	86.5	81.8
G4P6/Po/GBR/F	82.6	82.9	83.6	81.6	83.9	86.5	86	86.5	83.9	84.7	86.5	86	86.5	84.1	84.9	82.9	83.9	82.3	87	82.3
G4P6/Po/BRA/BRA844-07	88.6	82.3	84.7	81.8	90.4	86.5	86.5	87.8	83.6	83.4	88.3	86.2	87.8	83.6	87	82.3	90.1	82.3	88	82.3
G4P6/Po/GBR/VLAB	88.6	82.3	83.4	78.7	89.1	83.6	85.2	84.4	84.4	83.1	84.9	84.4	85.4	83.9	84.4	82.3	89.1	83.6	84.7	83.6
G4P6/Po/JPN/pig6-7d	80.3	79.7	80	81.6	80.3	79	79.2	78.4	77.7	76.6	78.4	77.4	78.7	77.7	80	79.7	80.8	76.1	79	76.1

RVA strain	G3P6/Po/ POL/551	G4P6/Po/ POL/825	G4P6/Po/ POL/1306	G4P6/Po/ POL/1372	G4P6/Po/ POL/1373	G4P6/Po/ POL/921	G4P6/Po/ POL/923	G4P6/Po/ POL/1185	G4P6/Po/ POL/1194	G4P6/Po/ POL/874	G5P6/Po/ POL/450	G4P6/Po/ POL/597	G4P6/Po/ POL/870	G4P6/Po/ POL/871	G4P6/Po/ POL/1225	G4P6/Po/ POL/790	G4P6/Po/ POL/139	G9P6/Po/ POL/546	G5P6/Po/ POL/733	G4P6/Hu/ POL/188
G4P6/Hu/GBR/ST3	81.8	81.8	88.8	84.4	84.4	82.3	82.3	83.4	82.9	82.9	88.8	83.6	81.3	81.3	82.6	81.3	90.4	81.3	83.4	76.1
G4P6/Po/POL/53	84.4	84.4	81.8	83.4	83.4	86	86	83.9	85.4	86	81.3	82.1	84.4	84.4	83.6	83.9	81.8	83.4	83.9	79
G4P6/Po/POL/1046	84.7	84.7	83.6	84.1	84.1	86.7	86.7	86.7	85.7	86.7	83.1	83.4	84.1	84.1	84.9	86.2	83.6	85.4	86.7	77.9
G5P6/Po/POL/1075	78.7	78.7	79.7	79.5	79.5	80.5	80.5	83.4	80.3	79.7	79.2	78.4	81.3	81.3	82.3	81.6	78.7	81.8	83.4	87
G5P6/Po/POL/449	83.4	83.4	99.4	84.7	84.7	85.2	85.2	83.9	86.2	85.2	100	83.4	83.6	83.6	83.1	82.6	91.4	82.3	83.9	77.9
G4P6/Po/POL/1224	85.7	85.7	83.6	84.1	84.1	86	86	94.5	85.2	86.2	83.1	83.9	83.9	83.9	100	93.5	85.2	94.3	94.5	81.3
G4P6/Po/POL/786	85.4	85.4	83.1	84.4	84.4	85.2	85.2	95.8	85.4	86.5	82.6	83.1	83.6	83.6	93.5	100	85.7	95	95.8	82.6
G4P6/Po/POL/962	84.7	84.7	83.9	85.2	85.2	85.7	85.7	96.6	85.4	85.2	83.4	84.9	83.9	83.9	94	94.8	84.7	95.5	96.6	81.3
G4P6/Po/POL/920	95.3	95.3	85.4	84.1	84.1	99.7	99.7	86.5	94.8	96.3	84.9	82.6	94.8	94.8	85.7	85.4	84.7	84.4	86.5	78.2
G4P6/Po/POL/868	93.7	93.7	83.6	83.6	83.6	95	95	84.7	95.5	94.3	83.6	82.1	100	100	83.9	83.6	83.1	83.1	84.7	78.7
G4P6/Po/POL/1099	85.7	85.7	84.1	85.2	85.2	85.4	85.4	96.6	86.2	86.2	83.6	84.9	83.9	83.9	94.8	95	86	95.8	96.6	81
G5P6/Po/POL/857	84.4	84.4	82.9	84.1	84.1	84.7	84.7	96.6	84.9	85.4	82.3	84.4	83.1	83.1	94.3	95	84.9	100	96.6	80.8
G9P6/Po/POL/775	86	86	84.4	86	86	86.7	86.7	100	86.5	87	83.9	85.7	84.7	84.7	94.5	95.8	86.5	96.6	100	82.1
G9P6/Po/POL/1040	95.5	95.5	86	83.9	83.9	96.8	96.8	85.7	94.5	96.6	85.4	82.3	94.5	94.5	86	84.7	84.9	84.1	85.7	79.2
G5P6/Po/POL/620	82.1	82.1	84.7	100	100	84.4	84.4	86	85.2	83.6	84.7	96.8	83.6	83.6	84.1	84.4	84.9	84.1	86	80
G9P6/Po/POL/38	84.4	84.4	81.8	83.4	83.4	86	86	83.9	85.4	86	81.3	82.1	84.4	84.4	83.6	83.9	81.8	83.4	83.9	79
G9P6/Po/POL/1307	83.9	83.9	99.7	84.7	84.7	85.7	85.7	84.4	86.2	85.7	99.2	83.4	83.6	83.6	83.6	83.1	91.4	82.9	84.4	78.2
G3P6/Po/POL/823	100	100	83.9	82.1	82.1	95.5	95.5	86	92.2	96.3	83.4	81	93.7	93.7	85.7	85.4	84.9	84.4	86	77.7
G4P6/Po/POL/964	84.9	84.9	84.1	85.4	85.4	86	86	97.1	86	85.4	83.6	85.2	84.1	84.1	94.5	95.3	85.2	96.1	97.1	81.6
G4P6/Po/POL/822	100	100	83.9	82.1	82.1	95.5	95.5	86	92.2	96.3	83.4	81	93.7	93.7	85.7	85.4	84.9	84.4	86	77.7
G3P6/Po/POL/551	-	100	83.9	82.1	82.1	95.5	95.5	86	92.2	96.3	83.4	81	93.7	93.7	85.7	85.4	84.9	84.4	86	77.7
G4P6/Po/POL/825	100	-	83.9	82.1	82.1	95.5	95.5	86	92.2	96.3	83.4	81	93.7	93.7	85.7	85.4	84.9	84.4	86	77.7
G4P6/Po/POL/1306	83.9	83.9	-	84.7	84.7	85.7	85.7	84.4	86.2	85.7	99.4	83.4	83.6	83.6	83.6	83.1	91.4	82.9	84.4	78.4
G4P6/Po/POL/1372	82.1	82.1	84.7	-	100	84.4	84.4	86	85.2	83.6	84.7	96.8	83.6	83.6	84.1	84.4	84.9	84.1	86	80
G4P6/Po/POL/1373	82.1	82.1	84.7	100	-	84.4	84.4	86	85.2	83.6	84.7	96.8	83.6	83.6	84.1	84.4	84.9	84.1	86	80
G4P6/Po/POL/921	95.5	95.5	85.7	84.4	84.4	-	100	86.7	95	96.6	85.2	82.9	95	95	86	85.2	84.9	84.7	86.7	78.2
G4P6/Po/POL/923	95.5	95.5	85.7	84.4	84.4	100	-	86.7	95	96.6	85.2	82.9	95	95	86	85.2	84.9	84.7	86.7	78.2
G4P6/Po/POL/1185	86	86	84.4	86	86	86.7	86.7	-	86.5	87	83.9	85.7	84.7	84.7	94.5	95.8	86.5	96.6	100	82.1
G4P6/Po/POL/1194	92.2	92.2	86.2	85.2	85.2	95	95	86.5	-	93.7	86.2	83.6	95.5	95.5	85.2	85.4	85.7	84.9	86.5	79
G4P6/Po/POL/874	96.3	96.3	85.7	83.6	83.6	96.6	96.6	87	93.7	-	85.2	82.1	94.3	94.3	86.2	86.5	85.7	85.4	87	78.7
G5P6/Po/POL/450	83.4	83.4	99.4	84.7	84.7	85.2	85.2	83.9	86.2	85.2	-	83.4	83.6	83.6	83.1	82.6	91.4	82.3	83.9	77.9
G4P6/Po/POL/597	81	81	83.4	96.8	96.8	82.9	82.9	85.7	83.6	82.1	83.4	-	82.1	82.1	83.9	83.1	84.1	84.4	85.7	79.2
G4P6/Po/POL/870	93.7	93.7	83.6	83.6	83.6	95	95	84.7	95.5	94.3	83.6	82.1	-	100	83.9	83.6	83.1	83.1	84.7	78.7
G4P6/Po/POL/871	93.7	93.7	83.6	83.6	83.6	95	95	84.7	95.5	94.3	83.6	82.1	100	-	83.9	83.6	83.1	83.1	84.7	78.7
G4P6/Po/POL/1225	85.7	85.7	83.6	84.1	84.1	86	86	94.5	85.2	86.2	83.1	83.9	83.9	83.9	-	93.5	85.2	94.3	94.5	81.3
G4P6/Po/POL/790	85.4	85.4	83.1	84.4	84.4	85.2	85.2	95.8	85.4	86.5	82.6	83.1	83.6	83.6	93.5	-	85.7	95	95.8	82.6
G4P6/Po/POL/139	84.9	84.9	91.4	84.9	84.9	84.9	84.9	86.5	85.7	85.7	91.4	84.1	83.1	83.1	85.2	85.7	-	84.9	86.5	77.9
G9P6/Po/POL/546	84.4	84.4	82.9	84.1	84.1	84.7	84.7	96.6	84.9	85.4	82.3	84.4	83.1	83.1	94.3	95	84.9	-	96.6	80.8
G5P6/Po/POL/733	86	86	84.4	86	86	86.7	86.7	100	86.5	87	83.9	85.7	84.7	84.7	94.5	95.8	86.5	96.6	-	82.1
G4P6/Hu/POL/188	77.7	77.7	78.4	80	80	78.2	78.2	82.1	79	78.7	77.9	79.2	78.7	78.7	81.3	82.6	77.9	80.8	82.1	-
G4P6/Po/CHE/S19-1116	89.3	89.3	82.9	83.9	83.9	91.4	91.4	85.7	90.6	90.9	82.3	83.9	89.8	89.8	84.1	83.9	82.3	84.4	85.7	77.4
G4P6/Po/CHE/S19-1115	89.3	89.3	82.9	83.9	83.9	91.4	91.4	85.7	90.6	90.9	82.3	83.9	89.8	89.8	84.1	83.9	82.3	84.4	85.7	77.4
G4P6/Po/HRV/S243-VS	90.6	90.6	85.4	84.9	84.9	92.4	92.4	88	92.2	92.2	84.9	83.4	91.7	91.7	86.2	87	84.4	87	88	78.7
G4P6/Po/SVK/TOPC28	79.2	79.2	79.5	77.7	77.7	79.7	79.7	83.6	79.5	79.5	79	76.6	80.5	80.5	82.1	82.3	78.4	82.1	83.6	86.2
G4P6/Po/SVK/LSKC30	91.7	91.7	85.7	85.4	85.4	93.7	93.7	87.3	92.4	92.7	85.2	83.9	92.2	92.2	85.4	86.2	84.9	85.7	87.3	77.9
G4P6/Po/SVK/LSKC25	91.9	91.9	85.4	85.7	85.7	94	94	87.5	92.2	93	84.9	84.1	92.4	92.4	85.7	86.5	84.7	86	87.5	78.2
G4P6/Po/SVK/LSKC24	91.9	91.9	85.4	85.7	85.7	94	94	87.5	92.2	93	84.9	84.1	92.4	92.4	85.7	86.5	84.7	86	87.5	78.2
G4P6/Hu/ITA/PZ3	82.6	82.6	93.2	87.5	87.5	84.1	84.1	83.4	84.7	83.6	93.2	86.2	82.6	82.6	84.1	83.1	90.9	82.9	83.4	79.2
G4P6/Po/HRV/S400-VS	82.6	82.6	83.9	87.3	87.3	83.9	83.9	85.4	84.7	84.7	83.9	86.5	85.2	85.2	85.7	84.9	83.6	84.7	85.4	81.6
G4P6/Po/HRV/S397-VS	82.6	82.6	83.9	87.3	87.3	83.9	83.9	85.4	84.7	84.7	83.9	86.5	85.2	85.2	85.7	84.9	83.6	84.7	85.4	81.6
G5P6/Po/SVN/P83	91.7	91.7	86.2	86.2	86.2	94.8	94.8	88.3	93.5	93.2	85.7	84.7	93	93	86.7	86.5	84.9	86.5	88.3	79.7
G4P6/Hu/HUN/BP1125	91.7	91.7	85.2	85.2	85.2	94	94	87	93.2	93.2	84.7	83.6	93	93	85.7	86.5	84.1	86	87	78.7
G4P6/Hu/HUN/BP1547	89.6	89.6	86	84.9	84.9	91.7	91.7	86	90.6	91.1	85.4	83.6	90.6	90.6	84.7	85.4	83.9	85.7	86	77.9
G5P6/Po/GBR/X1	81.8	81.8	83.4	85.2	85.2	83.6	83.6	86.5	86.2	83.4	83.4	84.9	84.1	84.1	86	85.4	86	85.4	86.5	81
G4P6/Po/GBR/F	82.3	82.3	83.9	84.9	84.9	84.1	84.1	86.5	86.2	83.9	83.9	84.7	84.7	84.7	86.5	86	86.2	86	86.5	80.5
G4P6/Po/BRA/BRA844-07	82.3	82.3	90.4	87	87	83.9	83.9	87.8	84.9	83.4	90.4	85.7	83.4	83.4	86.5	86.5	90.1	86.2	87.8	81.3
G4P6/Po/GBR/VLAB	83.6	83.6	89.1	84.4	84.4	84.7	84.7	85.4	84.7	84.7	89.1	83.6	83.1	83.1	83.6	85.2	90.9	84.4	85.4	78.2
G4P6/Po/JPN/pig6-7d	76.1	76.1	80.8	80	80	77.7	77.7	78.7	78.4	78.2	80.3	78.7	76.6	76.6	79	79.2	80.3	77.4	78.7	82.1

RVA strain	G4P6/Po/CHE/S19-1116	G4P6/Po/CHE/S19-1115	G4P6/Po/HRV/S243-VS	G4P6/Po/SVK/TOPC28	G4P6/Po/SVK/LSKC30	G4P6/Po/SVK/LSKC25	G4P6/Po/SVK/LSKC24	G4P6/Hu/ITA/PZ3	G4P6/Po/HRV/S400-VS	G4P6/Po/HRV/S397-VS	G5P6/Po/SVN/P83	G4P6/Hu/HUN/BP1125	G4P6/Hu/HUN/BP1547	G5P6/Po/GBR/X1	G4P6/Po/GBR/F	G4P6/Po/BRA/BRA844-07
G4P6/Hu/GBR/ST3	79.7	79.7	82.6	76.9	82.6	82.3	82.3	89.3	83.4	83.4	83.9	82.9	82.6	82.6	82.6	88.6
G4P6/Po/POL/53	85.4	85.4	85.4	79.5	84.4	84.7	84.7	81.3	84.9	84.9	86.2	85.4	84.1	82.3	82.9	82.3
G4P6/Po/POL/1046	86.2	86.2	86.2	80.5	85.7	86	86	83.1	84.4	84.4	87	86.7	86.2	83.1	83.6	84.7
G5P6/Po/POL/1075	82.1	82.1	81.6	96.6	80	80.3	80.3	80.3	82.3	82.3	82.3	80.5	80	80.8	81.6	81.8
G5P6/Po/POL/449	82.3	82.3	84.9	79	85.2	84.9	84.9	93.2	83.9	83.9	85.7	84.7	85.4	83.4	83.9	90.4
G4P6/Po/POL/1224	84.1	84.1	86.2	82.1	85.4	85.7	85.7	84.1	85.7	85.7	86.7	85.7	84.7	86	86.5	86.5
G4P6/Po/POL/786	83.9	83.9	87	82.3	86.2	86.5	86.5	83.1	84.9	84.9	86.5	86.5	85.4	85.4	86	86.5
G4P6/Po/POL/962	85.7	85.7	87.3	84.1	86.5	86.7	86.7	84.9	84.7	84.7	87.8	86.7	85.7	86	86.5	87.8
G4P6/Po/POL/920	91.1	91.1	92.2	79.7	93.5	93.7	93.7	83.9	83.6	83.6	94.5	93.7	91.4	83.4	83.9	83.6
G4P6/Po/POL/868	89.8	89.8	91.7	80.5	92.2	92.4	92.4	82.6	85.2	85.2	93	93	90.6	84.1	84.7	83.4
G4P6/Po/POL/1099	84.7	84.7	86.7	82.1	86	86.2	86.2	84.1	86.2	86.2	87.3	86.2	85.2	86	86.5	88.3
G5P6/Po/POL/857	84.4	84.4	87	82.1	85.7	86	86	82.9	84.7	84.7	86.5	86	85.7	85.4	86	86.2
G9P6/Po/POL/775	85.7	85.7	88	83.6	87.3	87.5	87.5	83.4	85.4	85.4	88.3	87	86	86.5	86.5	87.8
G9P6/Po/POL/1040	90.6	90.6	92.4	79.5	93	92.7	92.7	84.4	83.4	83.4	93.5	94	91.9	83.6	84.1	83.6
G5P6/Po/POL/620	83.9	83.9	84.9	77.7	85.4	85.7	85.7	87.5	87.3	87.3	86.2	85.2	84.9	85.2	84.9	87
G9P6/Po/POL/38	85.4	85.4	85.4	79.5	84.4	84.7	84.7	81.3	84.9	84.9	86.2	85.4	84.1	82.3	82.9	82.3
G9P6/Po/POL/1307	82.9	82.9	85.4	79.2	85.7	85.4	85.4	93	83.6	83.6	86.2	85.2	86	83.4	83.9	90.1
G3P6/Po/POL/823	89.3	89.3	90.6	79.2	91.7	91.9	91.9	82.6	82.6	82.6	91.7	91.7	89.6	81.8	82.3	82.3
G4P6/Po/POL/964	86	86	87.5	84.4	86.7	87	87	85.2	84.9	84.9	88	87	86	86.5	87	88
G4P6/Po/POL/822	89.3	89.3	90.6	79.2	91.7	91.9	91.9	82.6	82.6	82.6	91.7	91.7	89.6	81.8	82.3	82.3
G3P6/Po/POL/551	89.3	89.3	90.6	79.2	91.7	91.9	91.9	82.6	82.6	82.6	91.7	91.7	89.6	81.8	82.3	82.3
G4P6/Po/POL/825	89.3	89.3	90.6	79.2	91.7	91.9	91.9	82.6	82.6	82.6	91.7	91.7	89.6	81.8	82.3	82.3
G4P6/Po/POL/1306	82.9	82.9	85.4	79.5	85.7	85.4	85.4	93.2	83.9	83.9	86.2	85.2	86	83.4	83.9	90.4
G4P6/Po/POL/1372	83.9	83.9	84.9	77.7	85.4	85.7	85.7	87.5	87.3	87.3	86.2	85.2	84.9	85.2	84.9	87
G4P6/Po/POL/1373	83.9	83.9	84.9	77.7	85.4	85.7	85.7	87.5	87.3	87.3	86.2	85.2	84.9	85.2	84.9	87
G4P6/Po/POL/921	91.4	91.4	92.4	79.7	93.7	94	94	84.1	83.9	83.9	94.8	94	91.7	83.6	84.1	83.9
G4P6/Po/POL/923	91.4	91.4	92.4	79.7	93.7	94	94	84.1	83.9	83.9	94.8	94	91.7	83.6	84.1	83.9
G4P6/Po/POL/1185	85.7	85.7	88	83.6	87.3	87.5	87.5	83.4	85.4	85.4	88.3	87	86	86.5	86.5	87.8
G4P6/Po/POL/1194	90.6	90.6	92.2	79.5	92.4	92.2	92.2	84.7	84.7	84.7	93.5	93.2	90.6	86.2	86.2	84.9
G4P6/Po/POL/874	90.9	90.9	92.2	79.5	92.7	93	93	83.6	84.7	84.7	93.2	93.2	91.1	83.4	83.9	83.4
G5P6/Po/POL/450	82.3	82.3	84.9	79	85.2	84.9	84.9	93.2	83.9	83.9	85.7	84.7	85.4	83.4	83.9	90.4
G4P6/Po/POL/597	83.9	83.9	83.4	76.6	83.9	84.1	84.1	86.2	86.5	86.5	84.7	83.6	83.6	84.9	84.7	85.7
G4P6/Po/POL/870	89.8	89.8	91.7	80.5	92.2	92.4	92.4	82.6	85.2	85.2	93	93	90.6	84.1	84.7	83.4
G4P6/Po/POL/871	89.8	89.8	91.7	80.5	92.2	92.4	92.4	82.6	85.2	85.2	93	93	90.6	84.1	84.7	83.4
G4P6/Po/POL/1225	84.1	84.1	86.2	82.1	85.4	85.7	85.7	84.1	85.7	85.7	86.7	85.7	84.7	86	86.5	86.5
G4P6/Po/POL/790	83.9	83.9	87	82.3	86.2	86.5	86.5	83.1	84.9	84.9	86.5	86.5	85.4	85.4	86	86.5
G4P6/Po/POL/139	82.3	82.3	84.4	78.4	84.9	84.7	84.7	90.9	83.6	83.6	84.9	84.1	83.9	86	86.2	90.1
G9P6/Po/POL/546	84.4	84.4	87	82.1	85.7	86	86	82.9	84.7	84.7	86.5	86	85.7	85.4	86	86.2
G5P6/Po/POL/733	85.7	85.7	88	83.6	87.3	87.5	87.5	83.4	85.4	85.4	88.3	87	86	86.5	86.5	87.8
G4P6/Hu/POL/188	77.4	77.4	78.7	86.2	77.9	78.2	78.2	79.2	81.6	81.6	79.7	78.7	77.9	81	80.5	81.3
G4P6/Po/CHE/S19-1116	-	100	90.4	81	88.8	89.1	89.1	82.9	83.6	83.6	90.6	90.4	88.3	83.4	83.9	83.6
G4P6/Po/CHE/S19-1115	100	-	90.4	81	88.8	89.1	89.1	82.9	83.6	83.6	90.6	90.4	88.3	83.4	83.9	83.6
G4P6/Po/HRV/S243-VS	90.4	90.4	-	82.3	93.7	94	94	84.4	84.9	84.9	94.5	94.8	92.7	84.7	85.2	85.2
G4P6/Po/SVK/TOPC28	81	81	82.3	-	79.7	80	80	80	81	81	81.6	80.3	79.7	79.5	80	81
G4P6/Po/SVK/LSKC30	88.8	88.8	93.7	79.7	-	99.7	99.7	84.4	84.1	84.1	94.8	95.8	94	86	86.5	85.2
G4P6/Po/SVK/LSKC25	89.1	89.1	94	80	99.7	-	100	84.1	84.4	84.4	95	96.1	94.3	85.7	86.2	84.9
G4P6/Po/SVK/LSKC24	89.1	89.1	94	80	99.7	100	-	84.1	84.4	84.4	95	96.1	94.3	85.7	86.2	84.9
G4P6/Hu/ITA/PZ3	82.9	82.9	84.4	80	84.4	84.1	84.1	-	83.6	83.6	84.7	84.7	84.7	84.4	84.9	90.4
G4P6/Po/HRV/S400-VS	83.6	83.6	84.9	81	84.1	84.4	84.4	83.6	-	100	86	84.1	83.9	84.7	84.9	87
G4P6/Po/HRV/S397-VS	83.6	83.6	84.9	81	84.1	84.4	84.4	83.6	100	-	86	84.1	83.9	84.7	84.9	87
G5P6/Po/SVN/P83	90.6	90.6	94.5	81.6	94.8	95	95	84.7	86	86	-	95.5	93.7	85.4	86	86
G4P6/Hu/HUN/BP1125	90.4	90.4	94.8	80.3	95.8	96.1	96.1	84.7	84.1	84.1	95.5	-	96.6	84.4	84.9	84.9
G4P6/Hu/HUN/BP1547	88.3	88.3	92.7	79.7	94	94.3	94.3	84.7	83.9	83.9	93.7	96.6	-	82.9	83.4	84.1
G5P6/Po/GBR/X1	83.4	83.4	84.7	79.5	86	85.7	85.7	84.4	84.7	84.7	85.4	84.4	82.9	-	98.9	85.7
G4P6/Po/GBR/F	83.9	83.9	85.2	80	86.5	86.2	86.2	84.9	84.9	84.9	86	84.9	83.4	98.9	-	86.2
G4P6/Po/BRA/BRA844-07	83.6	83.6	85.2	81	85.2	84.9	84.9	90.4	87	87	86	84.9	84.1	85.7	86.2	-
G4P6/Po/GBR/VLAB	81.8	81.8	84.4	78.4	85.4	85.2	85.2	89.1	84.1	84.1	86.2	84.7	84.7	83.9	84.4	89.8
G4P6/Po/JPN/pig6-7d	77.9	77.9	78.7	80.5	78.7	79	79	80.8	82.3	82.3	79	78.2	77.7	79.2	79.2	82.3

RVA strain	G4P6/Po/GBR/VLAB	G4P6/Po/JPN/pig6-7d
G4P6/Hu/GBR/ST3	88.6	80.3
G4P6/Po/POL/53	82.3	79.7
G4P6/Po/POL/1046	83.4	80
G5P6/Po/POL/1075	78.7	81.6
G5P6/Po/POL/449	89.1	80.3
G4P6/Po/POL/1224	83.6	79
G4P6/Po/POL/786	85.2	79.2
G4P6/Po/POL/962	84.4	78.4
G4P6/Po/POL/920	84.4	77.7
G4P6/Po/POL/868	83.1	76.6
G4P6/Po/POL/1099	84.9	78.4
G5P6/Po/POL/857	84.4	77.4
G9P6/Po/POL/775	85.4	78.7
G9P6/Po/POL/1040	83.9	77.7
G5P6/Po/POL/620	84.4	80
G9P6/Po/POL/38	82.3	79.7
G9P6/Po/POL/1307	89.1	80.8
G3P6/Po/POL/823	83.6	76.1
G4P6/Po/POL/964	84.7	79
G4P6/Po/POL/822	83.6	76.1
G3P6/Po/POL/551	83.6	76.1
G4P6/Po/POL/825	83.6	76.1
G4P6/Po/POL/1306	89.1	80.8
G4P6/Po/POL/1372	84.4	80
G4P6/Po/POL/1373	84.4	80
G4P6/Po/POL/921	84.7	77.7
G4P6/Po/POL/923	84.7	77.7
G4P6/Po/POL/1185	85.4	78.7
G4P6/Po/POL/1194	84.7	78.4
G4P6/Po/POL/874	84.7	78.2
G5P6/Po/POL/450	89.1	80.3
G4P6/Po/POL/597	83.6	78.7
G4P6/Po/POL/870	83.1	76.6
G4P6/Po/POL/871	83.1	76.6
G4P6/Po/POL/1225	83.6	79
G4P6/Po/POL/790	85.2	79.2
G4P6/Po/POL/139	90.9	80.3
G9P6/Po/POL/546	84.4	77.4
G5P6/Po/POL/733	85.4	78.7
G4P6/Hu/POL/188	78.2	82.1
G4P6/Po/CHE/S19-1116	81.8	77.9
G4P6/Po/CHE/S19-1115	81.8	77.9
G4P6/Po/HRV/S243-VS	84.4	78.7
G4P6/Po/SVK/TOPC28	78.4	80.5
G4P6/Po/SVK/LSKC30	85.4	78.7
G4P6/Po/SVK/LSKC25	85.2	79
G4P6/Po/SVK/LSKC24	85.2	79
G4P6/Hu/ITA/PZ3	89.1	80.8
G4P6/Po/HRV/S400-VS	84.1	82.3
G4P6/Po/HRV/S397-VS	84.1	82.3
G5P6/Po/SVN/P83	86.2	79
G4P6/Hu/HUN/BP1125	84.7	78.2
G4P6/Hu/HUN/BP1547	84.7	77.7
G5P6/Po/GBR/X1	83.9	79.2
G4P6/Po/GBR/F	84.4	79.2
G4P6/Po/BRA/BRA844-07	89.8	82.3
G4P6/Po/GBR/VLAB	-	79.2
G4P6/Po/JPN/pig6-7d	79.2	-
