# Supplementary material for: Detection of Porcine–Human Reassortant and Zoonotic Group A Rotaviruses in Humans in Poland
Source: Transbound Emerg Dis. 2024 Sep 24;2024:4232389. doi: 10.1155/2024/4232389 (PMC12017087; doi:10.1155/2024/4232389)
Supplement: Supporting Information S6 — Table 6: the nucleotide sequence similarity of the VP4 gene fragment of pig and human P[8] RVA strains. [file 4232389.f6.pdf]

Supplementary Table S6. The nucleotide sequence similarity of the VP4 gene fragment of pig and human P[8] RVA strains

**RVA strain**	G1P8/Hu/USA/Wa	G1P8/Hu/IND/mani-375	G1P8/Hu/HUN/ERN5611	G1P8/Hu/BEL/BE1141	G9P8/Hu/SVN/SI-744	G1P8/Hu/FRA/E10585	G1P8/Hu/AUS/CK00110	G1P8/Hu/RUS/NN725-14	G4P8/Hu/ITA/PR1602	G1P8/Hu/POL/82	G1P8/Hu/POL/133	G1P8/Hu/POL/370
G1P8/Hu/USA/Wa	-	90.3	90.3	85.4	90.6	98.0	90.9	90.3	90.5	90.6	90.6	89.9
G1P8/Hu/IND/mani-375	90.3	-	96.2	85.3	96.7	89.8	98.1	98.1	96.3	96.5	96.5	97.6
G1P8/Hu/HUN/ERN5611	90.3	96.2	-	85.0	96.5	90,0	97.2	97.2	96.0	99.7	99.7	96.3
G1P8/Hu/BEL/BE1141	85.4	85.3	85,0	-	85.6	85.3	85.4	85.4	85.7	85.3	85.3	85.2
G9P8/Hu/SVN/SI-744	90.6	96.7	96.5	85.6	-	90.3	97.2	97.2	98.7	96.7	96.7	96.6
G1P8/Hu/FRA/E10585	98,0	89.8	90.0	85.3	90.3	-	90.6	90.0	90.2	90.3	90.3	89.6
G1P8/Hu/AUS/CK00110	90.9	98.1	97.2	85.4	97.2	90.6	-	98.8	96.7	97.4	97.4	98.0
G1P8/Hu/RUS/NN725-14	90.3	98.1	97.2	85.4	97.2	90.0	98.8	-	96.7	97.4	97.4	98.0
G4P8/Hu/ITA/PR1602	90.5	96.3	96,0	85.7	98.7	90.2	96.7	96.7	-	96.3	96.3	96.2
G1P8/Hu/POL/82	90.6	96.5	99.7	85.3	96.7	90.3	97.4	97.4	96.3	-	100.0	96.6
G1P8/Hu/POL/133	90.6	96.5	99.7	85.3	96.7	90.3	97.4	97.4	96.3	100.0	-	96.6
G1P8/Hu/POL/370	89.9	97.6	96.3	85.2	96.6	89.6	98.0	98.0	96.2	96.6	96.6	-
G1P8/Hu/POL/31	90.9	96.5	96.2	86.0	98.8	90.6	96.9	96.9	99.5	96.5	96.5	96.3
G1P8/Hu/POL/38	90.6	96.7	96.5	85.6	100.0	90.3	97.2	97.2	98.7	96.7	96.7	96.6
G1P8/Hu/POL/121	90.6	96.3	96.0	85.6	98.7	90.3	97.0	96.7	99.1	96.3	96.3	96.2
G1P8/Hu/POL/260	90.2	97.9	96.6	85.4	96.9	89.9	98.3	98.3	96.5	96.9	96.9	99.7
G1P8/Hu/POL/160	90.0	96.2	95.9	85.9	98.8	89.8	96.6	96.6	98.1	96.2	96.2	96.0
G1P8/Hu/POL/166	90.3	97.4	96.5	85.9	96.7	90.0	98.1	98.1	96.3	96.7	96.7	99.0
G1P8/Hu/POL/193	89.9	96.3	96,0	85.7	98.7	89.6	96.7	96.7	98.0	96.3	96.3	96.2
G1P8/Hu/POL/176	90.0	97.7	96.5	85.3	96.7	89.8	98.1	98.1	96.3	96.7	96.7	99.8
G1P8/Hu/POL/180	90.9	96.3	96.0	85.6	98.7	90.6	96.7	96.7	99.4	96.3	96.3	96.2
G1P8/Hu/POL/254	90.3	98.0	96.5	85.3	97.0	90.0	98.1	98.1	96.6	96.7	96.7	99.5
G1P8/Hu/POL/255	90.2	97.9	96.3	85.4	96.9	89.9	98.0	98.0	96.5	96.6	96.6	99.7
G1P8/Hu/POL/257	90.0	97.7	96.5	85.3	96.7	89.8	98.1	98.1	96.3	96.7	96.7	99.8
G1P8/Hu/POL/262	90.0	97.7	96.5	85.3	96.7	89.8	98.1	98.1	96.3	96.7	96.7	99.8
G1P8/Hu/POL/274	90.0	97.7	96.5	85.3	96.7	89.8	98.1	98.1	96.3	96.7	96.7	99.8
G1P8/Hu/POL/104	90.6	96.7	96.5	85.6	100.0	90.3	97.2	97.2	98.7	96.7	96.7	96.6
G1P8/Hu/POL/109	90.2	96.9	97.0	85.3	96.7	89.8	97.6	97.9	96.0	97.3	97.3	96.7
G1P8/Hu/POL/114	90.6	97.4	96.5	85.6	96.7	90.3	98.1	98.1	96.3	96.7	96.7	99.0
G1P8/Hu/POL/316	90.5	97.2	96.2	85.4	96.5	90.5	97.9	97.9	96.3	96.5	96.5	98.7
G1P8/Hu/POL/324	90.2	97.0	96.0	85.7	96.3	89.9	97.7	97.7	95.9	96.3	96.3	98.6
G1P8/Hu/POL/330	89.9	97.6	96.3	85.2	96.6	89.6	98.0	98.0	96.2	96.6	96.6	99.7
G1P8/Hu/POL/248	90.0	96.7	96.6	85.2	96.6	89.6	97.4	97.7	95.9	96.9	96.9	96.6
G1P8/Hu/POL/302	90.0	96.7	96.9	85.2	96.6	89.6	97.4	97.7	95.9	97.2	97.2	96.6
G1P8/Hu/POL/305	89.9	97.6	96.3	85.2	96.6	89.6	98.0	98.0	96.2	96.6	96.6	99.7
G1P8/Hu/POL/308	90.0	97.7	96.5	85.3	96.7	89.8	98.1	98.1	96.3	96.7	96.7	99.8
G1P8/Hu/POL/335	90.0	96.9	95.9	85.0	96.5	89.8	97.6	97.6	96.0	96.2	96.2	98.4
G1P8/Hu/POL/345	90.2	97.0	96,0	85.2	96.6	89.9	97.7	97.7	96.2	96.3	96.3	98.6
G1P8/Po/POL/1160	89.8	96.0	95.8	85.9	98.4	89.5	96.5	96.5	97.7	96.0	96.0	95.9
G1P8/Po/HRV/S441-OB	90.3	96.5	95.9	85.4	99.1	90.0	96.9	96.9	98.1	96.2	96.2	96.3
G1P8/Hu/SVK/2764	90.0	97.7	96.5	85.3	96.7	89.8	98.1	98.1	96.3	96.7	96.7	99.8
G1P8/Po/HRV/S372-VS	89.8	95.6	95.3	85.3	98.3	89.5	96.0	96,0	97.6	95.6	95.6	95.5

**RVA strain**	G1P8/Hu/ POL/31	G1P8/Hu/ POL/38	G1P8/Hu/ POL/121	G1P8/Hu/ POL/260	G1P8/Hu/ POL/160	G1P8/Hu/ POL/166	G1P8/Hu/ POL/193	G1P8/Hu/ POL/176	G1P8/Hu/ POL/180	G1P8/Hu/ POL/254	G1P8/Hu/ POL/255	G1P8/Hu/ POL/257	G1P8/Hu/ POL/262	G1P8/Hu/ POL/274
G1P8/Hu/USA/Wa	90.9	90.6	90.6	90.2	90.0	90.3	89.9	90.0	90.9	90.3	90.2	90.0	90.0	90.0
G1P8/Hu/IND/mani-375	96.5	96.7	96.3	97.9	96.2	97.4	96.3	97.7	96.3	98.0	97.9	97.7	97.7	97.7
G1P8/Hu/HUN/ERN5611	96.2	96.5	96.0	96.6	95.9	96.5	96.0	96.5	96.0	96.5	96.3	96.5	96.5	96.5
G1P8/Hu/BEL/BE1141	86.0	85.6	85.6	85.4	85.9	85.9	85.7	85.3	85.6	85.3	85.4	85.3	85.3	85.3
G9P8/Hu/SVN/SI-744	98.8	100.0	98.7	96.9	98.8	96.7	98.7	96.7	98.7	97.0	96.9	96.7	96.7	96.7
G1P8/Hu/FRA/E10585	90.6	90.3	90.3	89.9	89.8	90.0	89.6	89.8	90.6	90.0	89.9	89.8	89.8	89.8
G1P8/Hu/AUS/CK00110	96.9	97.2	97.0	98.3	96.6	98.1	96.7	98.1	96.7	98.1	98.0	98.1	98.1	98.1
G1P8/Hu/RUS/NN725-14	96.9	97.2	96.7	98.3	96.6	98.1	96.7	98.1	96.7	98.1	98.0	98.1	98.1	98.1
G4P8/Hu/ITA/PR1602	99.5	98.7	99.1	96.5	98.1	96.3	98,0	96.3	99.4	96.6	96.5	96.3	96.3	96.3
G1P8/Hu/POL/82	96.5	96.7	96.3	96.9	96.2	96.7	96.3	96.7	96.3	96.7	96.6	96.7	96.7	96.7
G1P8/Hu/POL/133	96.5	96.7	96.3	96.9	96.2	96.7	96.3	96.7	96.3	96.7	96.6	96.7	96.7	96.7
G1P8/Hu/POL/370	96.3	96.6	96.2	99.7	96.0	99,0	96.2	99.8	96.2	99.5	99.7	99.8	99.8	99.8
G1P8/Hu/POL/31	-	98.8	99.3	96.6	98.3	96.5	98.1	96.5	99.5	96.7	96.6	96.5	96.5	96.5
G1P8/Hu/POL/38	98.8	-	98.7	96.9	98.8	96.7	98.7	96.7	98.7	97.0	96.9	96.7	96.7	96.7
G1P8/Hu/POL/121	99.3	98.7	-	96.5	98.1	96.3	98.0	96.3	99.1	96.6	96.5	96.3	96.3	96.3
G1P8/Hu/POL/260	96.6	96.9	96.5	-	96.3	99.3	96.5	99.8	96.5	99.5	99.7	99.8	99.8	99.8
G1P8/Hu/POL/160	98.3	98.8	98.1	96.3	-	96.2	99.8	96.2	98.1	96.5	96.3	96.2	96.2	96.2
G1P8/Hu/POL/166	96.5	96.7	96.3	99.3	96.2	-	96.3	99.1	96.3	98.8	99.0	99.1	99.1	99.1
G1P8/Hu/POL/193	98.1	98.7	98.0	96.5	99.8	96.3	-	96.3	98.0	96.6	96.5	96.3	96.3	96.3
G1P8/Hu/POL/176	96.5	96.7	96.3	99.8	96.2	99.1	96.3	-	96.3	99.7	99.8	100.0	100.0	100.0
G1P8/Hu/POL/180	99.5	98.7	99.1	96.5	98.1	96.3	98.0	96.3	-	96.6	96.5	96.3	96.3	96.3
G1P8/Hu/POL/254	96.7	97.0	96.6	99.5	96.5	98.8	96.6	99.7	96.6	-	99.8	99.7	99.7	99.7
G1P8/Hu/POL/255	96.6	96.9	96.5	99.7	96.3	99,0	96.5	99.8	96.5	99.8	-	99.8	99.8	99.8
G1P8/Hu/POL/257	96.5	96.7	96.3	99.8	96.2	99.1	96.3	100.0	96.3	99.7	99.8	-	100.0	100.0
G1P8/Hu/POL/262	96.5	96.7	96.3	99.8	96.2	99.1	96.3	100.0	96.3	99.7	99.8	100.0	-	100.0
G1P8/Hu/POL/274	96.5	96.7	96.3	99.8	96.2	99.1	96.3	100.0	96.3	99.7	99.8	100.0	100.0	-
G1P8/Hu/POL/104	98.8	100.0	98.7	96.9	98.8	96.7	98.7	96.7	98.7	97.0	96.9	96.7	96.7	96.7
G1P8/Hu/POL/109	96.2	96.7	96.0	97.0	96.2	96.9	96.3	96.9	96.0	96.9	96.7	96.9	96.9	96.9
G1P8/Hu/POL/114	96.5	96.7	96.3	99.3	96.2	99.4	96.3	99.1	96.3	98.8	99.0	99.1	99.1	99.1
G1P8/Hu/POL/316	96.5	96.5	96.3	99.0	95.9	99.1	96.0	98.8	96.3	98.6	98.7	98.8	98.8	98.8
G1P8/Hu/POL/324	96.0	96.3	95.9	98.8	95.8	99.0	95.9	98.7	95.9	98.4	98.6	98.7	98.7	98.7
G1P8/Hu/POL/330	96.3	96.6	96.2	99.7	96.0	99.0	96.2	99.8	96.2	99.5	99.7	99.8	99.8	99.8
G1P8/Hu/POL/248	96.0	96.6	95.9	96.9	96.0	96.7	96.2	96.7	95.9	96.7	96.6	96.7	96.7	96.7
G1P8/Hu/POL/302	96.0	96.6	95.9	96.9	96.0	96.7	96.2	96.7	95.9	96.7	96.6	96.7	96.7	96.7
G1P8/Hu/POL/305	96.3	96.6	96.2	99.7	96.0	99,0	96.2	99.8	96.2	99.5	99.7	99.8	99.8	99.8
G1P8/Hu/POL/308	96.5	96.7	96.3	99.8	96.2	99.1	96.3	100.0	96.3	99.7	99.8	100.0	100.0	100.0
G1P8/Hu/POL/335	96.2	96.5	96.0	98.7	95.9	98.8	96.0	98.6	96.0	98.3	98.4	98.6	98.6	98.6
G1P8/Hu/POL/345	96.3	96.6	96.2	98.8	96.0	99.0	96.2	98.7	96.2	98.4	98.6	98.7	98.7	98.7
G1P8/Po/POL/1160	97.9	98.4	97.7	96.2	**99.5**	96.0	**99.4**	96.0	97.7	96.3	96.2	96.0	96.0	96.0
G1P8/Po/HRV/S441-OB	98.6	99.1	98.1	96.6	98.3	96.5	98.1	96.5	98.1	96.7	96.6	96.5	96.5	96.5
G1P8/Hu/SVK/2764	96.5	96.7	96.3	99.8	96.2	99.1	96.3	100.0	96.3	99.7	99.8	100.0	100.0	100.0
G1P8/Po/HRV/S372-VS	97.7	98.3	97.6	95.8	99.4	95.6	99.3	95.6	97.6	95.9	95.8	95.6	95.6	95.6

**RVA strain**	G1P8/Hu/POL/114	G1P8/Hu/POL/316	G1P8/Hu/POL/324	G1P8/Hu/POL/330	G1P8/Hu/POL/248	G1P8/Hu/POL/302	G1P8/Hu/POL/305	G1P8/Hu/POL/308	G1P8/Hu/POL/335	G1P8/Hu/POL/345	G1P8/Po/POL/1160	G1P8/Po/HRV/S441-OB	G1P8/Hu/SVK/2764	G1P8/Po/HRV/S372-VS
G1P8/Hu/USA/Wa	90.6	90.5	90.2	89.9	90.0	90.0	89.9	90.0	90.0	90.2	89.8	90.3	90.0	89.8
G1P8/Hu/IND/mani-375	97.4	97.2	97.0	97.6	96.7	96.7	97.6	97.7	96.9	97.0	96.0	96.5	97.7	95.6
G1P8/Hu/HUN/ERN5611	96.5	96.2	96.0	96.3	96.6	96.9	96.3	96.5	95.9	96.0	95.8	95.9	96.5	95.3
G1P8/Hu/BEL/BE1141	85.6	85.4	85.7	85.2	85.2	85.2	85.2	85.3	85.0	85.2	85.9	85.4	85.3	85.3
G9P8/Hu/SVN/SI-744	96.7	96.5	96.3	96.6	96.6	96.6	96.6	96.7	96.5	96.6	98.4	99.1	96.7	98.3
G1P8/Hu/FRA/E10585	90.3	90.5	89.9	89.6	89.6	89.6	89.6	89.8	89.8	89.9	89.5	90,0	89.8	89.5
G1P8/Hu/AUS/CK00110	98.1	97.9	97.7	98.0	97.4	97.4	98.0	98.1	97.6	97.7	96.5	96.9	98.1	96.0
G1P8/Hu/RUS/NN725-14	98.1	97.9	97.7	98.0	97.7	97.7	98.0	98.1	97.6	97.7	96.5	96.9	98.1	96.0
G4P8/Hu/ITA/PR1602	96.3	96.3	95.9	96.2	95.9	95.9	96.2	96.3	96,0	96.2	97.7	98.1	96.3	97.6
G1P8/Hu/POL/82	96.7	96.5	96.3	96.6	96.9	97.2	96.6	96.7	96.2	96.3	96.0	96.2	96.7	95.6
G1P8/Hu/POL/133	96.7	96.5	96.3	96.6	96.9	97.2	96.6	96.7	96.2	96.3	96.0	96.2	96.7	95.6
G1P8/Hu/POL/370	99.0	98.7	98.6	99.7	96.6	96.6	99.7	99.8	98.4	98.6	95.9	96.3	99.8	95.5
G1P8/Hu/POL/31	96.5	96.5	96.0	96.3	96.0	96.0	96.3	96.5	96.2	96.3	97.9	98.6	96.5	97.7
G1P8/Hu/POL/38	96.7	96.5	96.3	96.6	96.6	96.6	96.6	96.7	96.5	96.6	98.4	99.1	96.7	98.3
G1P8/Hu/POL/121	96.3	96.3	95.9	96.2	95.9	95.9	96.2	96.3	96.0	96.2	97.7	98.1	96.3	97.6
G1P8/Hu/POL/260	99.3	99.0	98.8	99.7	96.9	96.9	99.7	99.8	98.7	98.8	96.2	96.6	99.8	95.8
G1P8/Hu/POL/160	96.2	95.9	95.8	96.0	96.0	96.0	96.0	96.2	95.9	96.0	**99.5**	98.3	96.2	99.4
G1P8/Hu/POL/166	99.4	99.1	99.0	99.0	96.7	96.7	99.0	99.1	98.8	99.0	96.0	96.5	99.1	95.6
G1P8/Hu/POL/193	96.3	96.0	95.9	96.2	96.2	96.2	96.2	96.3	96.0	96.2	**99.4**	98.1	96.3	99.3
G1P8/Hu/POL/176	99.1	98.8	98.7	99.8	96.7	96.7	99.8	100.0	98.6	98.7	96.0	96.5	100.0	95.6
G1P8/Hu/POL/180	96.3	96.3	95.9	96.2	95.9	95.9	96.2	96.3	96.0	96.2	97.7	98.1	96.3	97.6
G1P8/Hu/POL/254	98.8	98.6	98.4	99.5	96.7	96.7	99.5	99.7	98.3	98.4	96.3	96.7	99.7	95.9
G1P8/Hu/POL/255	99.0	98.7	98.6	99.7	96.6	96.6	99.7	99.8	98.4	98.6	96.2	96.6	99.8	95.8
G1P8/Hu/POL/257	99.1	98.8	98.7	99.8	96.7	96.7	99.8	100.0	98.6	98.7	96.0	96.5	100.0	95.6
G1P8/Hu/POL/262	99.1	98.8	98.7	99.8	96.7	96.7	99.8	100.0	98.6	98.7	96.0	96.5	100.0	95.6
G1P8/Hu/POL/274	99.1	98.8	98.7	99.8	96.7	96.7	99.8	100.0	98.6	98.7	96.0	96.5	100.0	95.6
G1P8/Hu/POL/104	96.7	96.5	96.3	96.6	96.6	96.6	96.6	96.7	96.5	96.6	98.4	99.1	96.7	98.3
G1P8/Hu/POL/109	96.9	96.6	96.7	96.7	99.5	99.8	96.7	96.9	96.3	96.5	96.0	96.2	96.9	95.6
G1P8/Hu/POL/114	-	99.4	99.3	99.0	96.7	96.7	99.0	99.1	99.1	99.3	96.0	96.5	99.1	95.6
G1P8/Hu/POL/316	99.4	-	99.0	98.7	96.5	96.5	98.7	98.8	99.1	99.3	95.8	96.2	98.8	95.6
G1P8/Hu/POL/324	99.3	99.0	-	98.6	96.6	96.6	98.6	98.7	98.7	98.8	95.6	96.0	98.7	95.2
G1P8/Hu/POL/330	99.0	98.7	98.6	-	96.6	96.6	100.0	99.8	98.4	98.6	95.9	96.3	99.8	95.5
G1P8/Hu/POL/248	96.7	96.5	96.6	96.6	-	99.7	96.6	96.7	96.2	96.3	95.9	96.0	96.7	95.5
G1P8/Hu/POL/302	96.7	96.5	96.6	96.6	99.7	-	96.6	96.7	96.2	96.3	95.9	96.0	96.7	95.5
G1P8/Hu/POL/305	99.0	98.7	98.6	100.0	96.6	96.6	-	99.8	98.4	98.6	95.9	96.3	99.8	95.5
G1P8/Hu/POL/308	99.1	98.8	98.7	99.8	96.7	96.7	99.8	-	98.6	98.7	96.0	96.5	100.0	95.6
G1P8/Hu/POL/335	99.1	99.1	98.7	98.4	96.2	96.2	98.4	98.6	-	99.3	95.8	96.2	98.6	95.8
G1P8/Hu/POL/345	99.3	99.3	98.8	98.6	96.3	96.3	98.6	98.7	99.3	-	95.9	96.3	98.7	95.8
G1P8/Po/POL/1160	96.0	95.8	95.6	95.9	95.9	95.9	95.9	96.0	95.8	95.9	-	97.9	96.0	99.0
G1P8/Po/HRV/S441-OB	96.5	96.2	96.0	96.3	96.0	96.0	96.3	96.5	96.2	96.3	97.9	-	96.5	97.7
G1P8/Hu/SVK/2764	99.1	98.8	98.7	99.8	96.7	96.7	99.8	100.0	98.6	98.7	96.0	96.5	-	95.6
G1P8/Po/HRV/S372-VS	95.6	95.6	95.2	95.5	95.5	95.5	95.5	95.6	95.8	95.8	99.0	97.7	95.6	-
