# Supplementary material for: Detection of Porcine–Human Reassortant and Zoonotic Group A Rotaviruses in Humans in Poland
Source: Transbound Emerg Dis. 2024 Sep 24;2024:4232389. doi: 10.1155/2024/4232389 (PMC12017087; doi:10.1155/2024/4232389)
Supplement: Supporting Information S7 — Table 7: the amino acid sequence changes in the VP4 gene fragment of pig and human P[6] RVA strains. [file 4232389.f7.pdf]

Supplementary Table S7. The amino acid sequence changes in the VP4 gene fragment of pig and human P[6] RVA strains

RVA strain	Amino acid positions in VP4 gene fragment																																																																													
	47	52	54	64	67	81	82	85	88	90	92	93	94	95	96	98	101	102	106	107	108	109	111	114	115	116	117	119	120	123	128	129	132	133	136	137	138	139	143	149	150	151	152	153	156	161	163	168	180	185	187	192	194	195	197	198	199	200	201	202	203	204	205	206	210	216	219	222	229	235	241	245	246	249	252	257	258
**OQ982449/RVA/Pig-wt/POL/870/2014/G4P6**	**N**	**T**	**S**	**I**	**V**	**N**	**D**	**I**	**N**	**T**	**Q**	**K**	**I**	**I**	**L**	**G**	**R**	**T**	**V**	**A**	**I**	**L**	**I**	**N**	**T**	**A**	**S**	**V**	**R**	**T**	**T**	**R**	**T**	**I**	**N**	**S**	**S**	**E**	**F**	**N**	**A**	**G**	**A**	**E**	**H**	**T**	**D**	**G**	**H**	**N**	**T**	**T**	**S**	**N**	**P**	**D**	**I**	**E**	**T**	**V**	**V**	**H**	**T**	**D**	**I**	**S**	**K**	**I**	**I**	**I**	**S**	**V**	**T**	**R**	**V**	**I**	**I**
OQ982435/RVA/Pig-wt/POL/1046/2015/G4P6	S	.	.	V	.	.	.	.	.	.	.	T	V	V	.	.	G	.	.	.	.	.	.	.	V	N	N	S	.	.	I	K	.	V	.	.	N	R	.	.	.	N	.	.	.	.	.	A	.	.	.	S	.	D	A	N	.	K	.	.	I	.	.	.	.	.	.	V	M	.	.	.	.	.	.	.	.
ON600526/RVA/Human-wt/POL/188/2015/G4P6	S	.	.	V	I	.	.	.	.	V	K	G	V	V	.	.	.	.	.	.	.	I	.	H	L	T	.	D	.	.	M	K	.	V	S	.	D	K	.	.	.	S	.	D	.	.	.	.	.	H	.	.	.	.	.	.	.	.	V	.	I	.	A	E	.	A	N	.	M	V	.	I	.	.	.	.	.
MK239683/RVA/Pig-wt/POL/1075/2015/G5P6	G	N	G	V	.	.	.	.	.	A	K	G	V	V	F	.	.	.	.	.	.	I	.	H	V	S	.	D	.	I	V	K	.	V	S	.	D	K	.	.	.	N	V	D	.	.	.	.	.	H	.	.	.	.	.	N	V	.	A	.	I	.	.	E	.	.	N	.	M	V	.	I	.	.	.	.	.
MK239699/RVA/Pig-wt/POL/868/2015/G4P6	.	.	.	.	.	.	.	.	.	.	.	.	.	.	.	.	.	.	.	.	.	.	.	.	.	.	.	.	.	.	.	.	.	.	.	.	.	.	.	.	.	.	.	.	.	.	.	.	.	.	.	.	.	.	.	.	.	.	.	.	.	.	.	.	.	.	.	.	.	.	.	.	.	.	.	.	.
OQ982450/RVA/Pig-wt/POL/871/2014/G4P6	.	.	.	.	.	.	.	.	.	.	.	.	.	.	.	.	.	.	.	.	.	.	.	.	.	.	.	.	.	.	.	.	.	.	.	.	.	.	.	.	.	.	.	.	.	.	.	.	.	.	.	.	.	.	.	.	.	.	.	.	.	.	.	.	.	.	.	.	.	.	.	.	.	.	.	.	.
OQ982431/RVA/Pig-wt/POL/53/2013/G4P6	S	.	.	V	.	.	.	.	.	.	.	T	V	V	.	.	.	.	.	.	.	.	.	.	V	N	.	S	.	.	I	K	.	V	.	.	N	R	.	.	.	S	.	.	.	A	.	A	.	.	.	S	.	D	A	N	.	.	.	.	I	.	.	.	.	.	.	V	M	.	.	.	.	.	I	.	.
OQ982456/RVA/Pig-wt/POL/139/2011/G4P6	.	.	.	.	.	.	.	.	.	.	.	Q	.	V	.	S	K	I	.	.	L	.	V	.	V	T	N	S	.	.	.	K	.	V	.	T	N	K	.	S	.	S	.	.	.	.	E	.	C	H	I	S	.	.	S	E	V	.	.	.	I	.	V	E	.	.	S	.	M	.	T	.	.	.	I	V	.
OQ982448/RVA/Pig-wt/POL/597/2013/G4P6	S	.	.	M	.	.	.	.	S	.	.	Q	V	V	.	.	.	.	I	.	.	.	.	.	V	T	N	S	K	V	.	K	.	.	.	.	N	K	.	S	.	N	.	.	.	.	.	.	.	H	.	S	.	.	S	E	V	.	.	.	I	.	.	E	.	.	N	.	.	.	.	.	.	.	.	V	.
OQ982453/RVA/Pig-wt/POL/786/2014/G4P6	S	.	.	V	.	I	N	.	.	I	.	.	.	V	.	.	.	.	.	.	.	.	.	.	V	T	N	N	.	.	.	K	.	V	.	.	N	K	.	S	.	S	.	.	.	.	.	.	.	.	.	S	L	.	A	.	.	.	.	.	I	.	.	.	.	.	.	.	M	.	.	.	.	.	.	.	.
OQ982454/RVA/Pig-wt/POL/790/2014/G4P6	S	.	.	V	.	I	N	.	.	I	.	.	.	V	.	.	.	.	.	.	.	.	.	.	V	T	N	N	.	.	.	K	.	V	.	.	N	K	.	S	.	S	.	.	.	.	.	.	.	.	.	S	L	.	A	.	.	.	.	.	I	.	.	.	.	.	.	.	M	.	.	.	.	.	.	.	.
OQ982432/RVA/Pig-wt/POL/822/2014/G4P6	.	.	.	V	.	.	.	.	.	.	.	.	.	.	.	C	.	I	.	.	.	.	.	.	.	T	.	S	K	.	.	K	I	V	.	.	.	K	.	.	.	.	.	.	.	.	.	.	.	.	.	.	.	.	.	.	.	.	.	.	.	.	.	.	.	.	.	.	.	.	.	.	.	.	.	.	.
OQ982434/RVA/Pig-wt/POL/825/2014/G4P6	.	.	.	V	.	.	.	.	.	.	.	.	.	.	.	C	.	I	.	.	.	.	.	.	.	T	.	S	K	.	.	K	I	V	.	.	.	K	.	.	.	.	.	.	.	.	.	.	.	.	.	.	.	.	.	.	.	.	.	.	.	.	.	.	.	.	.	.	.	.	.	.	.	.	.	.	.
OQ982445/RVA/Pig-wt/POL/874/2014/G4P6	.	.	.	V	.	.	.	.	.	.	.	.	.	.	.	.	.	.	.	.	.	.	.	.	.	T	.	S	.	.	.	K	.	V	.	.	.	K	.	.	V	.	.	.	.	.	.	.	.	.	.	.	.	.	.	.	.	.	.	.	.	.	.	.	.	.	.	.	.	.	.	.	.	.	.	.	.
MK239700/RVA/Pig-wt/POL/920/2015/G4P6	.	.	.	V	.	.	.	.	.	.	.	.	.	.	.	.	.	.	.	.	.	.	.	.	.	I	.	S	.	.	.	K	I	V	.	.	.	K	.	.	.	.	.	.	.	.	.	.	.	.	.	S	.	.	.	.	.	.	.	.	.	.	A	.	.	.	.	.	.	.	.	.	.	.	.	.	.
OQ982441/RVA/Pig-wt/POL/921/2014/G4P6	.	.	.	V	.	.	.	.	.	.	.	.	.	.	.	.	.	.	.	.	.	.	.	.	.	I	.	S	.	.	.	K	I	V	.	.	.	K	.	.	.	.	.	.	.	.	.	.	.	.	.	S	.	.	.	.	.	.	.	.	.	.	A	.	.	.	.	.	.	.	.	.	.	.	.	.	.
OQ982442/RVA/Pig-wt/POL/923/2014/G4P6	.	.	.	V	.	.	.	.	.	.	.	.	.	.	.	.	.	.	.	.	.	.	.	.	.	I	.	S	.	.	.	K	I	V	.	.	.	K	.	.	.	.	.	.	.	.	.	.	.	.	.	S	.	.	.	.	.	.	.	.	.	.	A	.	.	.	.	.	.	.	.	.	.	.	.	.	.
MK239701/RVA/Pig-wt/POL/962/2015/G4P6	S	.	.	V	.	.	.	.	.	.	.	.	.	V	.	.	.	.	.	.	.	.	.	.	V	T	N	N	.	.	.	K	.	V	D	.	N	K	.	S	.	N	.	.	.	.	.	.	.	.	.	S	.	.	A	.	.	.	.	.	I	.	.	.	.	.	.	.	M	.	.	.	.	.	.	.	.
OQ982430/RVA/Pig-wt/POL/964/2014/G4P6	S	.	.	V	.	.	.	.	.	.	.	.	.	V	.	.	.	.	.	.	.	.	.	.	V	T	N	N	.	.	.	K	.	V	D	.	N	K	.	S	.	N	.	.	.	.	.	.	.	.	.	S	.	.	A	.	.	.	.	.	I	.	.	.	.	.	.	.	M	.	.	.	.	.	.	.	.
MK239702/RVA/Pig-wt/POL/1099/2015/G4P6	S	.	.	V	.	.	.	.	.	.	.	.	.	V	.	.	.	.	.	V	.	.	.	.	V	T	N	N	.	.	.	K	.	V	.	.	N	K	.	S	.	N	.	.	.	.	.	.	.	.	.	S	.	.	A	.	.	.	.	I	I	Y	.	.	.	.	.	.	M	.	.	.	A	.	.	.	.
OQ982443/RVA/Pig-wt/POL/1185/2015/G4P6	S	.	.	V	.	.	.	.	.	.	.	.	.	V	.	.	.	.	.	.	.	.	.	.	V	T	N	N	.	.	.	K	.	V	.	.	N	K	.	S	.	N	.	.	.	.	.	.	.	.	.	S	.	.	A	A	.	.	.	.	I	.	.	.	.	.	.	.	M	.	.	.	.	.	.	.	.
OQ982444/RVA/Pig-wt/POL/1194/2015/G4P6	.	.	.	.	.	.	.	.	.	.	.	.	.	.	.	.	.	.	.	.	.	.	.	.	.	T	.	D	.	.	.	K	.	.	.	.	.	K	.	S	.	S	.	.	.	I	.	.	.	.	.	.	.	.	.	.	.	.	.	.	.	.	.	.	.	.	.	.	.	.	.	.	.	.	.	.	.
OQ982451/RVA/Pig-wt/POL/1224/2015/G4P6	S	.	.	V	.	.	.	.	.	.	.	.	.	V	.	.	K	.	.	.	.	.	.	.	V	T	N	N	.	.	.	K	I	V	.	.	N	K	.	S	.	.	.	.	.	.	.	.	.	D	.	S	.	.	A	.	.	.	.	.	I	.	.	.	.	.	.	.	M	.	.	.	.	.	.	.	.
OQ982452/RVA/Pig-wt/POL/1225/2015/G4P6	S	.	.	V	.	.	.	.	.	.	.	.	.	V	.	.	K	.	.	.	.	.	.	.	V	T	N	N	.	.	.	K	I	V	.	.	N	K	.	S	.	.	.	.	.	.	.	.	.	D	.	S	.	.	A	.	.	.	.	.	I	.	.	.	.	.	.	.	M	.	.	.	.	.	.	.	.
OQ982437/RVA/Pig-wt/POL/1306/2015/G4P6	.	.	.	V	.	.	.	.	S	.	.	Q	.	V	.	S	K	.	.	.	L	.	V	.	V	T	N	S	.	.	.	K	.	V	.	T	N	K	Y	S	V	S	.	.	S	I	.	.	.	H	.	S	.	.	S	E	.	.	.	.	I	.	.	E	V	.	N	.	M	.	.	.	.	.	.	.	V
OQ982439/RVA/Pig-wt/POL/1372/2015/G4P6	S	.	.	.	.	.	.	.	S	.	.	Q	V	V	.	.	.	.	I	.	.	.	.	.	V	T	N	S	.	V	.	K	.	.	.	.	N	K	.	S	.	S	V	.	.	.	.	.	.	H	.	S	.	.	S	E	V	.	.	.	I	.	.	E	.	.	N	.	M	.	.	.	.	.	.	.	.
OQ982440/RVA/Pig-wt/POL/1373/2015/G4P6	S	.	.	.	.	.	.	.	S	.	.	Q	V	V	.	.	.	.	I	.	.	.	.	.	V	T	N	S	.	V	.	K	.	.	.	.	N	K	.	S	.	S	V	.	.	.	.	.	.	H	.	S	.	.	S	E	V	.	.	.	I	.	.	E	.	.	N	.	M	.	.	.	.	.	.	.	.
OQ982433/RVA/Pig-wt/POL/551/2015/G3P6	.	.	.	V	.	.	.	.	.	.	.	.	.	.	.	C	.	I	.	.	.	.	.	.	.	T	.	S	K	.	.	K	I	V	.	.	.	K	.	.	.	.	.	.	.	.	.	.	.	.	.	.	.	.	.	.	.	.	.	.	.	.	.	.	.	.	.	.	.	.	.	.	.	.	.	.	.
MK239668/RVA/Pig-wt/POL/823/2015/G3P6	.	.	.	V	.	.	.	.	.	.	.	.	.	.	.	C	.	I	.	.	.	.	.	.	.	T	.	S	K	.	.	K	I	V	.	.	.	K	.	.	.	.	.	.	.	.	.	.	.	.	.	.	.	.	.	.	.	.	.	.	.	.	.	.	.	.	.	.	.	.	.	.	.	.	.	.	.
OQ982446/RVA/Pig-wt/POL/449/2015/G5P6	.	.	.	.	.	.	.	M	S	.	.	Q	.	V	.	S	K	.	.	.	L	.	V	.	V	T	N	S	.	.	.	K	.	V	.	T	N	K	Y	S	V	S	.	.	S	I	.	.	.	H	.	S	.	.	S	E	V	.	.	.	I	.	.	E	.	.	N	.	M	.	.	.	H	.	.	V
OQ982447/RVA/Pig-wt/POL/450/2015/G5P6	.	.	.	.	.	.	.	M	S	.	.	Q	.	V	.	S	K	.	.	.	L	.	V	.	V	T	N	S	.	.	.	K	.	V	.	T	N	K	Y	S	V	S	.	.	S	I	.	.	.	H	.	S	.	.	S	E	V	.	.	.	I	.	.	E	.	.	N	.	M	.	.	.	H	.	.	V
MK239674/RVA/Pig-wt/POL/620/2013/G5P6	S	.	.	.	.	.	.	.	S	.	.	Q	V	V	.	.	.	.	I	.	.	.	.	.	V	T	N	S	.	V	.	K	.	.	.	.	N	K	.	S	.	S	V	.	.	.	.	.	.	H	.	S	.	.	S	E	V	.	.	.	I	.	.	E	.	.	N	.	M	.	.	.	.	.	.	.	.
MK239676/RVA/Pig-wt/POL/733/2014/G5P6	S	.	.	V	.	.	.	.	.	.	.	.	.	V	.	.	.	.	.	.	.	.	.	.	V	T	N	N	.	.	.	K	.	V	.	.	N	K	.	S	.	N	.	.	.	.	.	.	.	.	.	S	.	.	A	A	.	.	.	.	I	.	.	.	.	.	.	.	M	.	.	.	.	.	.	.	.
MK239677/RVA/Pig-wt/POL/857/2014/G5P6	S	.	.	V	.	.	.	.	.	.	.	.	.	V	.	.	.	.	.	.	.	.	.	.	V	T	N	N	.	.	.	K	.	V	.	.	N	K	.	S	.	S	.	.	.	.	.	.	.	.	.	S	.	.	A	.	.	.	.	.	.	.	.	.	.	.	.	.	M	.	.	.	.	.	.	.	.
OQ982455/RVA/Pig-wt/POL/38/2013/G9P6	S	.	.	V	.	.	.	.	.	.	.	T	V	V	.	.	.	.	.	.	.	.	.	.	V	N	.	S	.	.	I	K	.	V	.	.	N	R	.	.	.	S	.	.	.	A	.	A	.	.	.	S	.	D	A	N	.	.	.	.	I	.	.	.	.	.	.	V	M	.	.	.	.	.	I	.	.
OQ982457/RVA/Pig-wt/POL/546/2015/G9P6	S	.	.	V	.	.	.	.	.	.	.	.	.	V	.	.	.	.	.	.	.	.	.	.	V	T	N	N	.	.	.	K	.	V	.	.	N	K	.	S	.	S	.	.	.	.	.	.	.	.	.	S	.	.	A	.	.	.	.	.	.	.	.	.	.	.	.	.	M	.	.	.	.	.	.	.	.
MK239689/RVA/Pig-wt/POL/775/2014/G9P6	S	.	.	V	.	.	.	.	.	.	.	.	.	V	.	.	.	.	.	.	.	.	.	.	V	T	N	N	.	.	.	K	.	V	.	.	N	K	.	S	.	N	.	.	.	.	.	.	.	.	.	S	.	.	A	A	.	.	.	.	I	.	.	.	.	.	.	.	M	.	.	.	.	.	.	.	.
MK239690/RVA/Pig-wt/POL/1040/2015/G9P6	.	.	.	V	.	.	.	.	S	.	.	.	.	.	.	.	.	.	.	.	.	.	.	.	.	T	.	S	.	.	.	K	I	V	.	.	.	K	.	.	.	.	.	.	.	.	.	.	.	.	.	.	.	.	.	.	.	.	.	.	.	.	.	.	.	.	.	.	.	.	.	.	.	.	I	.	.
OQ982438/RVA/Pig-wt/POL/1307/2015/G9P6	.	.	.	V	.	.	.	.	S	.	.	Q	.	V	.	S	K	.	.	.	L	.	V	.	V	T	N	S	.	.	.	K	.	V	.	T	N	K	Y	S	V	S	.	.	S	I	.	.	.	H	.	S	.	.	S	E	.	.	.	.	I	.	.	E	V	.	N	.	M	.	.	.	.	.	.	.	V
