# Supplementary material for: Detection of Porcine–Human Reassortant and Zoonotic Group A Rotaviruses in Humans in Poland
Source: Transbound Emerg Dis. 2024 Sep 24;2024:4232389. doi: 10.1155/2024/4232389 (PMC12017087; doi:10.1155/2024/4232389)
Supplement: Supporting Information S8 — Table 8: the amino acid sequence changes in the VP4 gene fragment of pig and human P[8] RVA strains. [file 4232389.f8.pdf]

Supplementary Table S8. The amino acid sequence changes in the VP4 gene fragment of pig and human P[8] RVA strains

RVA strain	Amino acid positions in VP4 gene fragment																				
	30	31	60	72	73	78	85	104	113	130	144	149	162	173	187	194	195	230	234	245	248
**MK239665/RVA/Pig-wt/POL/1160/2015/G1P8**	**T**	**Q**	**T**	**T**	**A**	**T**	**T**	**A**	**N**	**V**	**K**	**N**	**R**	**V**	**S**	**D**	**G**	**R**	**P**	**K**	**Q**
ON600481/RVA/Human-wt/POL/82/2013/G9P8	.	.	.	.	T	.	N	.	D	.	R	.	.	I	.	N	.	.	.	.	.
ON600497/RVA/Human-wt/POL/133/2013/G9P8	.	.	.	.	T	.	N	.	D	.	R	.	.	I	.	N	.	.	.	.	.
ON600485/RVA/Human-wt/POL/370/2015/G1P8	.	.	.	.	T	.	N	.	D	.	R	S	K	I	.	N	.	.	.	T	.
ON600486/RVA/Human-wt/POL/31/2013/G4P8	.	.	.	.	T	.	.	.	.	.	R	.	.	.	.	.	.	.	.	.	.
ON600487/RVA/Human-wt/POL/38/2013/G9P8	.	.	.	.	T	.	.	.	.	.	R	.	.	.	.	.	.	.	.	.	.
ON600490/RVA/Human-wt/POL/121/2013/G1P8	.	.	.	.	T	.	.	.	.	I	R	.	.	.	G	.	.	.	.	.	.
ON600493/RVA/Human-wt/POL/260/2015/G1P8	.	.	.	.	T	.	N	.	D	.	R	S	K	I	.	N	.	.	.	T	.
ON600494/RVA/Human-wt/POL/160/2013/G1P8	.	.	.	.	.	.	.	.	.	.	.	.	.	.	.	.	.	.	.	.	.
ON600495/RVA/Human-wt/POL/166/2015/G1P8	.	.	.	.	T	.	N	V	D	.	R	S	K	I	.	N	D	.	.	T	.
ON600496/RVA/Human-wt/POL/193/2015/G3P8	.	.	.	.	.	.	.	.	.	.	.	.	.	I	.	.	.	.	.	.	.
ON600498/RVA/Human-wt/POL/176/2013/G1P8	.	.	.	.	T	.	N	.	D	.	R	S	K	I	.	N	.	.	.	T	.
ON600499/RVA/Human-wt/POL/180/2015/G9P8	.	.	.	.	T	.	.	.	.	.	R	.	.	.	.	.	.	.	.	.	.
ON600489/RVA/Human-wt/POL/254/2015/G1P8	.	.	.	.	T	.	N	.	.	.	R	.	K	I	.	N	.	.	.	T	.
ON600520/RVA/Human-wt/POL/255/2015/G1P8	.	.	.	.	T	.	N	.	.	.	R	S	K	I	.	N	.	.	.	T	.
ON600517/RVA/Human-wt/POL/257/2015/G1P8	.	.	.	.	T	.	N	.	D	.	R	S	K	I	.	N	.	.	.	T	.
ON600483/RVA/Human-wt/POL/262/2015/G1P8	.	.	.	.	T	.	N	.	D	.	R	S	K	I	.	N	.	.	.	T	.
ON600484/RVA/Human-wt/POL/274/2015/G1P8	.	.	.	.	T	.	N	.	D	.	R	S	K	I	.	N	.	.	.	T	.
ON600501/RVA/Human-wt/POL/104/2013/G9P8	.	.	.	.	T	.	.	.	.	.	R	.	.	.	.	.	.	.	.	.	.
ON600502/RVA/Human-wt/POL/109/2013/G9P8	.	.	.	.	T	S	N	.	D	.	R	.	.	I	.	N	.	.	.	.	.
ON600503/RVA/Human-wt/POL/114/2013/G1P8	.	.	.	.	T	.	N	.	D	.	R	S	K	I	.	N	D	.	.	T	.
ON600507/RVA/Human-wt/POL/316/2015/G4P8	.	.	.	.	T	.	N	.	D	.	R	S	K	I	.	N	D	.	.	T	.
ON600509/RVA/Human-wt/POL/324/2015/G1P8	.	.	.	A	T	.	N	.	D	.	R	S	K	I	.	N	D	.	.	T	.
ON600510/RVA/Human-wt/POL/330/2015/G1P8	I	.	.	.	T	.	N	.	D	.	R	S	K	I	.	N	.	.	.	T	.
ON600508/RVA/Human-wt/POL/248/2015/G1P8	.	K	.	.	T	S	N	.	D	.	R	.	.	I	.	N	.	.	.	.	.
ON600513/RVA/Human-wt/POL/302/2015/G1P8	.	.	.	.	T	S	N	.	D	.	R	.	.	I	.	N	.	.	.	.	.
ON600514/RVA/Human-wt/POL/305/2015/G1P8	I	.	.	.	T	.	N	.	D	.	R	S	K	I	.	N	.	.	.	T	.
ON600515/RVA/Human-wt/POL/308/2015/G1P8	.	.	.	.	T	.	N	.	D	.	R	S	K	I	.	N	.	.	.	T	.
ON600518/RVA/Human-wt/POL/335/2015/G4P8	.	.	.	.	T	.	N	.	D	.	R	S	K	I	.	N	D	.	A	T	E
ON600523/RVA/Human-wt/POL/345/2015/G4P8	.	.	A	.	T	.	N	.	D	.	R	S	K	I	.	N	D	I	.	T	.
